# From microbial proteins to cultivated meat for alternative meat-like products: a review on sustainable fermentation approaches

**DOI:** 10.1186/s13036-025-00509-9

**Published:** 2025-05-14

**Authors:** Fernando Roberto Paz Cedeno, Olumide Joseph Olubiyo, Sungil Ferreira

**Affiliations:** Department of Food Science, The University of ArkansasSystem – Division of Agriculture (UADA), , Fayetteville, AR 72704 USA

**Keywords:** Biomass fermentation, Environmental impact, Agricultural by-products, Bioenergy integration, Circular economy

## Abstract

The global demand for protein is rapidly increasing due to population growth and changing dietary preferences, highlighting the need for sustainable alternatives to traditional animal-based proteins. This review explores cultivated meat and microbial alternative proteins, focusing on their potential to meet nutritional needs while mitigating environmental impacts. It also examines the production of cultivated meat as well as various sources of microbial proteins, including mycoproteins, bacterial proteins, and microalgae, highlighting their nutritional profiles, production methods, and commercial applications. This includes an evaluation of the state of commercialization of mycoproteins and the innovative use of agricultural and industrial by-products as substrates for microbial fermentation. The integration of microbial protein production with the bioenergy sector is evaluated as a relevant alternative to attain a synergetic effect between energy and food production systems. Ultimately, this work aims to underscore the importance of microbial proteins in advancing towards a more sustainable protein production system, offering insights into current challenges and future opportunities in the field of fermentation to produce alternative proteins.

## Introduction

The global population has been steadily increasing, reaching 8 billion in 2022, with forecasts predicting it will approach 9.7 billion by 2050 [1]. To meet daily nutritional needs, 1.0 to 1.6 g of protein per kg of body weight, depending on the level of physical activity, is recommended [2]. The global meat production reached 333 million tons in 2018 [3], and it is expected to increase to 470 million tons annually by 2050 [4]. However, the environmental degradation caused by meat production is significant, contributing immensely to greenhouse gas emissions, land and water degradation, and toxicity from pesticides used in feed cultivation [5]. Meeting the food demands of this growing population requires innovative technologies and sustainable approaches to enhance agricultural productivity.

In 2024, global meat consumption remains substantial, with meat preferences varying across different global regions [6]. Total global meat consumption was projected to reach around 360 million tons in 2022, with countries like the United States and Australia showing the highest per capita consumption, while Southeast Asian nations consumed less animal-based protein [7]. However, the agricultural sector, particularly livestock production, is a major contributor to greenhouse gas emissions, land degradation, and inefficient use of natural resources, raising sustainability concerns for climate change [8, 9].

As a response, alternative protein sources like plant-based meat analogs, cultivated meat, and microbial proteins, such as mycoprotein proteins, have garnered significant attention as more sustainable protein options when compared to traditional meat from animal sources, being capable of addressing the growing demand for protein without jeopardizing natural resources on the way to meet those demands. Mycoproteins, derived from fungal biomass produced through aerobic fermentation, are recognized as a cost-effective, scalable, and consumer-preferred alternative [10, 11]. These protein ingredients are not only nutritious, high in protein, but also low in saturated fat and cholesterol, making them a healthier option while reducing the environmental footprint associated with traditional livestock farming [12]. In this sense, microbial sustainable protein alternatives represent a crucial innovation in addressing both global nutritional needs and environmental sustainability.

This scoping review aims to discuss alternative proteins for meat alternatives, specifically microbial proteins and cultivated meat. The review was conducted by systematically searching scientific literature without time constraints, with a focus on recent applications within the last 10 years. Inclusion criteria for scientific literature included peer-reviewed articles that discuss microbial proteins and cultivated meat. To understand and discuss industrial perspectives and international regulations, the search was expanded to include reports from governmental and non-governmental organizations, industry publications, and news outlets published within the last 5 years.

## Animal protein demand as food and its environmental impacts

Animal protein have long been a staple in human diets, offering a rich source of essential amino acids, vitamins and minerals. Traditional meat proteins are derived from livestock production, including cattle, poultry, pigs, and seafood. According to the Food and Agriculture Organization, livestock production and consumption have grown exponentially in recent decades [6, 7]. The world yearly production of meat increased from 70.563 million tons in 1961 to 355 million tons in 2022 with the Asian region emerging as the leading region in meat production, accounting for a significant share of the total global output. In 2023, China produced about 96.41 million metric tons of meat, of which 57.94 million metric tons of pork accounted for about 60% of national meat output, followed by poultry at 24.31 million metric tons accounting for about 20% and beef and veal at 8.62 million metric tons accounting for 8.9%. The United States, the second-largest meat producer and the largest poultry producer globally, produced 48 million metric tons of meat in 2023. Poultry meat (broiler chicken) accounted for 21 million metric tons, representing 44% of its total meat production that year. Pork, beef, and turkey comprised 26%, 25%, and 4% of the production, respectively, while other varieties such as lamb and mutton accounted for less than 1% [6, 13]. Production growth is in place to meet the growing meat demand, with the global consumption in 1961 and 2013 for beef and buffalo, pork, poultry and sheep and goat showing a 39.23, 87.5, 100.1 and 8.02 million tons increase respectively. This consumption growth is projected to continue with an expected 36% consumption increase in buffalo and beef, 21% for pork, 40% for poultry and 44% for sheep and goat by 2050 [6, 7].

Despite the functional and nutritional benefits of meat, its production and consumption have significant impacts on the environment, notably contributing to the greenhouse effect and other ecological challenges. The greenhouse gases emitted through livestock farming include methane (CH_4_), primarily from enteric fermentation in ruminants like cattle; nitrous oxide from manure management and fertilizer use for feed crops; and carbon dioxide (CO_2_) from deforestation and land use changes [14]. Methane, although short-lived compared to CO_2_, has a high global warming potential, with estimates suggesting it is 28 to 36 times more effective at trapping heat than CO_2_, over a 100-year period [15].

Beef/cattle production has been identified as one of the largest contributors to the livestock sector's environmental footprint, with emissions ranging from 27 to 60 kg CO₂-eq per kilogram of meat produced. This is due to methane emissions from enteric fermentation, extensive land use, and the long maturation period of cattle [14, 16]. Additionally, cattle ranching is a primary driver of deforestation, particularly in the Amazon region [17]. Pork production emits less greenhouse gases than beef but more than poultry, with emissions estimated between 7 and 12 kg CO₂-eq per kilogram of meat, mainly from feed production, manure management, and energy use in farming and processing [18]. Although pigs do not produce methane at the same level as ruminants, their concentrated waste systems still pose environmental risks. Poultry has the lowest greenhouse gases emissions of the three, with about 6 kg CO₂-eq per kilogram of chicken meat [19]. The shorter life cycle to slaughter and efficient feed conversion reduces emissions, but the scale of production can lead to substantial environmental impacts, particularly in terms of water and air pollution from concentrated animal feeding operations [20].

The mentioned environmental implications of meat consumption and production, combined with the specific functional properties that dictate meat quality, highlight the complex position of this food source in the modern world. In this sense, there is a need for sustainable meat production strategies as well as the exploration of meat alternatives that can balance nutritional needs, cultural preferences, expected sensorial properties and environmental impacts.

## Meat-like alternative proteins

Meat-like alternative proteins can be defined as proteins derived from non-traditional sources that can be obtained with low environmental impacts and provide a similar sensory experience as their animal counterpart. They can be classified based on their source such as plants, microbial, cultivated (meat) and microalgae [21]. In recent times, several of these proteins have been studied and developed for commercial use, employing modern extraction methods that highlight their potential as sustainable replacements for animal-based proteins.

In this scenario, plant-based protein products and ingredients spearheaded the work of educating consumers on alternative ways to have meat alternatives, penetrated and created their own market. Currently, plant-based protein leads the food industry as the most utilized alternative protein [22]. As ways to further advance the development of plant proteins products, efforts have been made to apply new technologies using novel extraction techniques like high-pressure processing, microwave-assisted extraction, enzymatic-assisted extraction and ultrasound-assisted extraction. However, challenges related to sensory attributes, allergenicity and functional properties have limited plant protein further growth and broader acceptance [23].

In general, consumers have a limited pre-existing knowledge of microalgae protein. However, consumers are open to consuming (no preconceptions) and acceptance of the consumption of microalgae protein has foundations on novelty, edibility, affordability, sustainability and healthiness [24]. The protein content in microalgae can vary from 30 to 70% in dry mass and this variation is based on different species, incubation and process conditions. In recent years, studies have been done to evaluate the functional properties of microalgae protein, such as emulsification properties, stability at different pH values and salt concentrations [25–28].

Cultivated meat, also known as lab-grown, cell-based, or cultured meat, is a rapid-growing concept in the food industry that involves producing authentic animal meat by cultivating animal cells, bypassing traditional farming methods [29]. This process aims to replicate the sensory, nutritional, and culinary characteristics of conventional meat in a more sustainable and ethical manner. Definitions from various organizations highlight the technological process of growing cells in bioreactors to develop meat without requiring an animal's full lifecycle, emphasizing its potential to reduce the environmental impact of meat production [30, 31]. Overall, cultivated meat represents a significant technological advancement and a shift towards more sustainable and humane food production practices.

Microbial protein consists of dried cells of microorganisms used in both animal and human nutrition [32]. It is highly nutritious, containing not only protein but also sugars, lipids, vitamins, minerals, and free amino acids [33, 34]. Microbial protein offers several advantages over plant-based protein, such as higher protein content, faster growth, and production, and independence from seasonal variations [35]. It can be cultivated on non-arable land and convert waste materials into valuable biomass, thus reducing greenhouse gas emissions and promoting waste valorization [33, 36, 37]. The rapid growth rate of these organisms allows for quick production cycles [38]. Microbial proteins are versatile, with applications ranging from human consumption and animal feed to biofuels and cosmetics, making them an attractive option for diverse industrial uses while supporting food security and environmental sustainability [39–41]. Main sources include microalgae, fungi, yeast, and bacteria, many of which are Generally Recognized as Safe (GRAS) [41].

Consumers consider a good meat-like alternative protein as one that closely replicates the sensory and functional characteristics of traditional meat while aligning with their dietary restrictions. Soy protein, a common ingredient in plant-based meat analogues, is especially valued for its high protein content, evidenced by a Protein Digestibility Corrected Amino Acid Score (PDCAAS) of 1, and for its functional versatility in creating meat-like textures [42]. Similarly, pea protein combined with wheat gluten has become prominent due to wheat gluten’s ability to enhance flavor and sensory qualities in meat analogs. However, concerns over allergies and sensitivities associated with soy and gluten-based meat-like products have limited their broader adoption and increase in consumption. Several studies have examined the protein digestibility of meat analogs compared to animal meat. In one of these studies, an *in-vitro* comparison of the protein digestibility and gastrointestinal behavior of meat analogs with that of beef was performed, finding that proteins in beef were more easily digested in the stomach than those in soy-based meat analogs [43]. Similarly, another study investigated the digestible indispensable amino acid score (DIAAS) and true ileal amino acid digestibility (TID) of plant-based proteins, comparing gel and emulsion properties. The study concluded that protein digestibility is influenced by both the protein source and processing methods. These differences can be attributed to the structural properties of plant-based meat-like products [44].

## Plant-based proteins

Over the last 20 years, plant proteins have spearheaded the path for alternative protein to gain consumers interest as well as market penetration. In general, some consumers perceive plant protein products as"freedom foods"due to their lower environmental impact and minimal health and safety concerns, when compared to their animal counterparts. The keyword"plant-based"has seen a rise over the past decade, being among the top three global food trends in 2020 [45, 46]. Currently, more than 90% of the plant protein market consists of soy, wheat, and pea protein [47]. However, wheat and soy are among the “Big 8” allergens [48]. Protein from cereals, pseudo cereals and legumes has been used around the world, contributing to promising nutritional and functional attributes [49, 50]. The use of plant protein as an ingredient has nutritional and marketing impacts, such “Clean Label”, “Vegan”, “(Good/Excellent) Source of Protein”, “Green” labels are effective ways to encourage the consumer to purchase plant protein-based products [51–55]. The main motivators driving purchasing habits of plant protein products are taste, cost, convenience, health and wellness, safety, environmental concern, animal welfare, and consumer familiarity with the protein source. On the other hand, the main consumer demotivators are health concerns, such as antinutritional factors and allergenicity, lack of familiarity, and desire for “meat-like” sensory experience. These motivators/demotivators are not absolute, since age, income, gender, education, and geographic location are also factors that influence consumers’ decisions [56]. Considering new techniques to improve functional properties and utilizing pulses instead of soy due to allergenicity concerns, previous studies evaluated the functional properties of protein concentrates from yellow pea, chickpea and lentil extracted using ultrafiltration and isoelectric precipitation methods [57]. Although these pulses showed high protein content yield and certain functional properties improvement (excluding water holding capacity), there were notable variations depending on the type of pulse. However, the functional properties and protein profile of these protein ingredients remained inferior to those of animal protein and soy proteins. Other current challenges faced when relying on plant proteins for further market advancement of alternative proteins include sensorial and functional challenges, low protein yield, and dependence on seasonality [35]. In this sense, there is a need for other sources of alternative proteins that, alongside plant proteins, can meet consumer demands and mitigate the current environmental impacts of animal protein production.

## Cultivated meats

Cultivated meat, often interchangeably referred to as lab-grown, cell-based, or cultured meat, has emerged as a groundbreaking concept in the food industry. According to the [29]. This approach aims to replicate the sensory, nutritional, and culinary characteristics of conventional meat in a more sustainable and ethical manner.

Cultivated meat can be defined as an animal protein produced by performing a biopsy from an animal, growing these cells in a bioreactor with a nutrient-rich medium, and allowing them to proliferate into tissues that resemble traditional meat in both structure and function [29]. This definition highlights the technological process by which cells are nurtured to develop into meat without requiring the full animal life cycle [58]. Similarly, the World Economic Forum describes cultivated meat as animal protein grown from animal cells in bioreactors, emphasizing its potential to replicate the appearance, taste, and texture of conventional meat while significantly reducing its environmental footprint [59]. This perspective underscores the sustainability-driven motivation behind the technology, aiming to lessen the ecological impact of meat production. Collectively, these definitions suggest that cultivated meat represents not only a technological advancement but also a shift towards more sustainable and humane food production practices.

The concept of growing meat outside an animal’s body has been considered since the early twentieth century, but it only became feasible in the early twenty-first century. These cells are then cultured in a nutrient-rich medium that resembles the body’s internal environment, promoting cell growth and division [60]. However, to achieve a meat-like structure, cells are often grown on scaffolds that provide a framework, which may be edible or biodegradable, or in bioreactors that regulate the growth environment, including temperature, pH, substrate and oxygen levels. The differentiation step is critical, as specific conditions stimulate precursor cells to develop into muscle, fat and connective tissues, which are essential for achieving the desired texture and flavor [61].

Beef is one of the earliest and most researched types of cultivated meats. This is largely due to the demand associated with it, as well as the environmental impact of cattle farming, which includes high greenhouse gas emissions and land use. Companies like Mosa Meat have pioneered beef cultivation, with the historic first'lab-grown burger'showcased in 2013. Previous works focused on the use of muscle satellite cells from cows to grow meat and explored growing fish and beef muscle in vitro, providing foundational knowledge for cultivating beef cells [60, 62, 63]. These works paved the way for advancing cultivated beef toward commercialization.

Chicken and other poultry products play a significant role in the cultivated meat sector, with Eat Just becoming one of the first companies to achieve regulatory approval for its cultured chicken product in Singapore in 2020. Recent studies explored optimizing the culture conditions of chicken satellite cells to improve growth rates and tissue formation [64], and the inhibition of p38 mitogen-activated protein kinase in chicken muscle stem cells to enhance their proliferation and differentiation during cultivation [65], significantly contributing to the knowledge of cultivated poultry.

Cultivated pork is gaining traction, with companies like Meatable leading the way, with works that investigated the potential of porcine stem cells to differentiate into edible muscle tissue on microcarriers, providing key insights into the biology of pork cultivation [66]. Additionally, the evaluation of the use of porcine-derived graft and the impact of scaffold materials on meat quality was described, contributing to the development of cultivated pork products [67]. Together, these studies are essential for refining techniques to produce lab-grown pork that can commercially and technologically compete with traditionally farmed meat.

Seafood and exotic meat cultivation, including the production of fish and shellfish, aims to address sustainability issues such as overfishing and pollution. A recent study evaluated the potential of cell-based fish production [68], while another study research has focused on the characterization of proliferation and differentiation for species like trout through in vitro assays to develop scalable seafood cultivation methods [69]. Although there is currently no well-established market for exotic meats, efforts are underway to produce exotic meat substitutes through cell cultivation, which are driven by both novelty and conservation motives. Efforts have been put into less common meat varieties as well, such as duck meat cells and its growth media requirements, and other less common species for meat production, with the goal of broadening the scope of cultivated meat and protecting wild species [63, 70].

Some companies blend cultivated meat with plant-based ingredients to create hybrid products that enhance sensory properties and reduce costs."GOOD Meat"by Eat Just exemplifies this approach [71]. Recent studies have explored the integration of cultivated meat cells with plant-based matrices to mimic the texture and nutritional profile of traditional meat products. In addition, the use of textured soy protein scaffolds in conjunction with bovine muscle tissue has been evaluated, aiming to achieve a more meat-like product through structural integration [72, 73]. These hybrid approaches could make cultivated meat more accessible and appeal to a broader consumer base, potentially accelerating its market acceptance.

The cultivated meat market is on the verge of a major transformation, with the potential to redefine meat consumption in a more sustainable, ethical manner that aligns with modern consumer values. Despite advancements, cultivated meat still faces research challenges, including cell type selection, growth media optimization, mass transfer issues in the bioreactor and the engineering of texture and flavor. Further research is essential to refining these technologies, ensuring that cultivated meat becomes a viable alternative to conventional meat in terms of taste, nutrition, and environmental impact [30, 74].

### Production of cultured meat

The production of cultured meat represents an innovative approach to creating meat products by cultivating animal cells in a controlled environment, eliminating the need for traditional animal slaughter. The process begins with cell sourcing, where a small biopsy is taken from an animal, such as a cow, chicken, or fish, to obtain stem cells or myoblasts capable of differentiating into muscle tissue [60]. These cells are then expanded in bioreactors, regulating environmental conditions like temperature, pH, and oxygen levels, providing a nutrient-rich medium containing amino acids, vitamins, minerals, and growth factors to promote cell proliferation [75]. Originally, fetal bovine serum (FBS) was a common component of this medium, but ethical and sustainability concerns have driven research toward serum-free alternatives [61]. Once sufficient cell numbers are achieved, differentiation is induced by altering the culture conditions to encourage the formation of muscle and fat cells, mimicking the composition of conventional meat. The final step involves structuring these cells into a meat-like product, often using edible scaffolds (like collagen) or advanced techniques such as 3D bioprinting to replicate the fibrous texture and layered structure of muscle tissue, as demonstrated by companies like Mosa Meat [76].

The extraction phase follows production, where the cultured meat is harvested from the bioreactors upon reaching the desired growth stage. This process varies depending on the production method: scaffold-based approaches require detaching the meat from its framework, while suspension cultures may involve centrifugation or filtration to collect the biomass [61]. Post-harvesting, the meat is processed to ensure safety and consumer readiness, which includes washing to remove residual culture medium, shaping it into forms like nuggets or steaks, and sometimes applying initial cooking or texturizing treatments. This step is critical to meet food safety standards and regulatory requirements, ensuring the product is free from contaminants and suitable for consumption, as outlined by agencies like the U.S. Department of Agriculture [77].

### Market and regulations of cultured meat

The market for cultivated meat is currently in its developing phase but is experiencing exponential growth. This sector is propelled by consumer demand for sustainable alternatives to traditional meat and by significant advancements in cellular agriculture technology. By 2023, the cultivated meat industry, though still niche, has attracted considerable investment, with billions of dollars invested into research and development over the last decade [78]. Market research suggests that the sector is expected to grow at a compound annual growth rate (CAGR) exceeding 15% in the coming years, potentially reaching multi-billion dollar valuations by 2030, driven by increased consumer acceptance, technological innovations and regulatory breakthroughs [79, 80].

Several companies are leading the cultivated meat industry, including Mosa Meat, Eat Just (with its GOOD Meat Brand), Aleph farms, UPSIDE Foods and BlueNalu. These companies focus on a variety of meat types, from beef and chicken to seafood and even exotic meats. The primary products introduced into the market so far include chicken nuggets and burgers. Mosa Meat has been considered a pioneer with cultivated beef, while BlueNalu focuses on high-value seafood products like bluefin tuna while Aleph Farms is targeting the steak market. Notably, GOOD Meat received the first regulatory approval for its chicken products in Singapore in 2020, followed by UPSIDE Foods and GOOD Meat gaining approval for chicken in the U.S in 2021 [81, 82].

Consumer acceptance of cultivated meat is multifaceted. There is enthusiasm among some consumers, particularly millennials and those concerned about sustainability, but skepticism persists due to unfamiliarity, the novel production method and concerns over costs and taste [83]. The industry is investing in consumer education to demystify the process and highlight the benefits of cultivated meat in terms of safety, ethics and environmental impact. For cultured meat to gain widespread acceptance, it must not only be safe but also deliver the sensory qualities of traditional meat [84].

The sector has seen robust investment from venture capitalists, corporations and even government bodies, underscoring a belief in the potential of cultivated meat to transform the food industry. This capital being directed towards research and development, particularly in the areas of bioreactor technology, cell culture media formulation and scaffold materials, which are crucial for reducing production costs and scaling operations [85]. Advances in 3D bioprinting and other scalable production methods are also pivotal for enabling the food industry to meet future demand [63].

Cultivated meat offers substantial environmental benefits, including reduced greenhouse gas emissions, less land and water use and the potential to leverage renewable energy sources for production [75]. From an ethical standpoint, it addresses concerns about animal welfare by producing meat without the need for animal slaughter, aligning with the growing consumer interest in more humane and sustainable protein sources [86]. However, navigating the regulatory environment is a critical aspect for companies in the cultivated meat sector. Singapore’s trailblazing approval set an international precedent, demonstrating that cultivated meat could meet stringent food safety standards [87]. The U.S has followed suit with selective approvals, highlighting the importance of safety allergenicity and nutritional equivalence to conventional meat products [31]. Nonetheless, the regulatory process varies significantly across countries presenting both challenges and opportunities for global market expansion. Companies must prove their products’ safety, nutritional value and compliance with labeling laws, making the approval a complex and time-consuming process.

Despite the promising outlook, challenges remain, particularly in scaling production to achieve price parity with conventional meat, which is essential for widespread consumer adoption. Moreover, while initial products have entered niche markets, achieving broader market penetration will require not only technological advancements but also strategic marketing, effective distribution channels and continued regulatory navigation [76].

### Limitations and opportunities of cultured meat

The production of cultivated meat encounters several limitations that impede its widespread adoption. Scalability remains a primary challenge, as current methods struggle to produce meat at a scale and cost-competitive with traditional livestock farming. Bioreactors, while effective for small-scale production, require significant scaling to meet global demand, which increases energy consumption and infrastructure costs [76]. Culture media cost is another hurdle,traditional media, such as those containing fetal bovine serum, are expensive and ethically contentious, while serum-free alternatives are still in development and often less efficient at promoting cell growth [75]. Regulatory uncertainty poses a barrier, with varying international standards necessitating extensive testing to prove safety, nutritional equivalence, and the absence of allergens, delaying market entry. Consumer acceptance is limited by skepticism regarding taste, texture, and the “unnatural” perception of lab-grown meat, requiring substantial efforts in education and sensory optimization [82]. Lastly, technical complexity in replicating the full structure of meat, including muscle, fat and connective tissues, adds layers of difficulty, as current products often lack the complexity of traditional cuts like steak [60].

Despite these limitations, cultivated meat benefits from numerous technological opportunities that could overcome current challenges. Bioreactor Innovation offers a pathway to scalability, with advancements in large-scale bioreactor design, such as perfusion systems and continuous culture technologies, enhancing cell density and reducing production costs. These innovations aim to mimic industrial fermentation processes, improving efficiency [88]. The development of serum-free media presents a significant opportunity, with research into upcycled agro-industrial plant-based or synthetic alternatives reducing reliance on FBS, lowering costs, and aligning with ethical production goals. Studies are exploring amino acid and growth factor formulations to optimize cell proliferation without animal-derived components [89]. Genetic engineering provides opportunities to enhance cell lines, enabling faster growth rates, increased resilience, or the ability to produce essential nutrients internally, potentially simplifying the culture process and reducing medium costs [90]. 3D bioprinting and scaffold technology are advancing to address structural complexity, allowing precise layering of muscle, fat, and connective tissues to mimic traditional meat more accurately. This technology could lead to products like cultivated steaks with improved texture and appeal [61]. Integration with plant-based Ingredients offers a hybrid approach, blending cultivated meat with plant proteins to enhance flavor, nutrition, and cost-effectiveness, as demonstrated by companies like GOOD Meat [71]. Finally, automation and artificial intelligence incorporated into production processes can optimize conditions in real-time, improving yield consistency and reducing labor costs, drawing parallels with precision fermentation advancements [91].

In summary, scalability, cost, regulatory hurdles, consumer acceptance and technical complexity currently are still challenges to the cultivated meat industry. However, innovations in bioreactor design, media development, genetic engineering, and structuring techniques offer pathways to overcome these barriers. As these technologies mature, cultivated meat has the potential to become a viable, sustainable alternative to conventional meat, reshaping the global food landscape while addressing environmental and ethical concerns.

## Microbial protein

Microbial protein consists of dried cells of microorganisms used in animal and human nutrition (e.g. mycoprotein) [32]. Despite its name, microbial protein is not exclusively composed of protein but has several important nutrients such as sugars, lipids, vitamins, minerals, and free amino acids [33, 34]. Microbial protein has been proven to be highly nutritious and to have an excellent amino acid profile, particularly highlighting lysine, methionine, and threonine, which makes microbial protein an excellent source for balanced human and animal nutrition, with a PDCAAS close to 1 [40].

Also, it offers several advantages when compared to plant-based protein, such as higher protein content, faster growth and production, and independence of seasonal variations [35]. They can be cultivated using non-arable land and can convert various waste materials into valuable microbial biomass. This includes CO_2_ for microalgae, agricultural by-products for fungi, and even methane or hydrogen for some bacteria, thus reducing greenhouse gas emissions and pollution while promoting waste valorization [33, 36, 37]. One of the most compelling advantages of microbial protein is the rapid growth rate of these organisms. Under optimal conditions, they can double their biomass in hours, allowing for quick production cycles and the potential for continuous or semi-continuous harvesting, which contrasts with the much slower growth of traditional protein sources like livestock or plant proteins [38].

The versatility of microbial proteins is also remarkable. Their applications span from direct human consumption (e.g., spirulina supplements or fungal-based meat alternatives) to animal feed, aquaculture, nutraceuticals, biofuels, and even the formulation of functional foods and cosmetics [39–41]. This adaptability makes microbial proteins an attractive option for diverse industrial applications, supporting both food security and environmental sustainability goals.

Microalgae, bacteria and fungi are the main sources of microbial protein, and many of these microorganisms are categorized as Generally Recognized as Safe (GRAS) [41].

### Microalgae protein

Microalgae represent a diverse group of photosynthetic organisms that are recognized for their potential for protein production. Several species have been studied for their high protein content and nutritional profile. *Arthrospira platensis (Spirulina)* is a renowned cyanobacterium known for its high protein content, often exceeding 60% on a dry-weight basis [92]. Spirulina is not only rich in essential amino acids but also contains a plethora of vitamins, minerals, and antioxidants, making it a superfood in the health food sector. *Chlorella vulgaris* is another well-studied microalga, containing protein levels around 50 to 60% [93]. It's particularly noted for its ability to enhance the immune system, detoxify heavy metals, and support digestive health due to its unique cell wall composition. *Nannochloropsis granulata* is recognized for its high protein content, which can reach up to 50% (dry weigh w/w), *Nannochloropsis* species are also valued for their significant lipid content, which is beneficial for both nutritional and biofuel applications [94]. This genus is particularly adaptable to various cultivation conditions, which contributes to its commercial viability.

The *Arthrospira* strain is utilized in human nutrition due to its high protein content and nutritional value. It can be used as a protein ingredient for the preparation of cookies, yogurt, and sausages [93, 95]. Additionally, this microalgae may offer several health benefits, including reducing high quality lipid levels, lowering blood pressure, protecting against kidney failure, acting as a prebiotic promoting the growth of beneficial intestinal bacteria such as *Lactobacillus*, and helping to control elevated serum glucose levels [96].

The protein content in microalgae, in general, ranges from 35 to 70% of their dry biomass, with the rest comprising carbohydrates (10 to 30%) and lipids (5 to 28%). This composition can vary based on factors like species, cultivation conditions (light, nutrients, temperature), and the growth phase. Numerous studies regarding the gross chemical composition of various algae have been documented in academic literature. To provide a comprehensive overview of the principal constituents, selected data pertaining to multiple micro-algal species have been compiled in Table 1.

**Table 1:** General composition of different microalgae (% of dry matter) and its applications

Source	Protein (%)	Lipid (%)	Carbohydrate (%)	Application	Reference
*Arthrospira maxima*	60–71	6–7	13–16	Meat sausages	[95, 97]
*Arthrospira platensis* (spirulina)	68.9	10.7	12.8	Yogurt, cookies	[92, 93]
*Chlorella vulgaris*	51–58	14–22	12–17	Cookies, Colorant, emulsifier, nutraceutical, animal feed	[93, 97–99]
*Tetraselmis suecica*	40.2	28.5	10.2	Cookies	[93]
*Phaeodactylum tricornutum*	38.8	19.3	11.0	Cookies	[93]
*Nannochloropsis granulata*	45.8	28.5	14.9	Animal feed	[94, 100]
*Nannochloropsis gaditana*	41.6	8.1	18.6	Colorant, cosmetics	[99, 101, 102]
*Nannochloropsis oculata*	42.1	15.6	16.7	Omega- 3 additive	[99]
*Phaeodactylum tricornutum*	39.0	14.9	15.4	Thickening, emulsifier	[99, 103]
*Scenedesmus obliquus*	50–56	12–14	10–17	Emulsifier	[97, 104]

### Bacterial protein

Bacteria form a diverse group of microorganisms known for their potential in microbial protein production. They possess the ability to thrive on a wide range of substrates, from simple sugars to complex industrial wastes, highlighting bacteria as a sustainable protein source and contributing to waste management while offering high-quality nutrition [105, 106]. Their role extends into various sectors of food technology, from protein supplements to innovative food products, showing the versatility of bacterial proteins in contemporary applications. Bacteria offer specific advantages such as rapid growth rate and high protein quality and content. Nevertheless, their high nucleic acid levels, reaching up to 16% (w/w) of dry weight, may render some of the bacterial protein inappropriate for human consumption [107].

Several bacterial species have been investigated for their high protein content and nutritional versatility. The adaptability of bacteria to different growth media and their rapid reproduction rates make them an attractive option for scalable and high-yield protein production. Moreover, the genetic manipulability of many bacterial strains allows for tailored protein production and decrease on nucleic acid levels, enhancing their application in both food and non-food industries. This positions bacterial proteins as a key player in addressing global protein demand sustainably. *Methylophilus methylotrophus*, for instance, is recognized for its use in producing single-cell protein (SCP) for animal feed, reaching up to 70% of protein content (dry weight) [108, 109]. Its ability to grow on methanol, a byproduct of industrial processes, exemplifies the sustainable aspect of bacterial SCP production. The proteins from this bacterium can support animal growth and health by providing a balanced amino acid profile, enhancing immune function, and potentially improving gut flora in animals. This indirectly benefits human health through safer and more nutritious animal products [110]. *Bacillus subtilis* is another bacterial species with significant protein content, around 40 to 50% [111], and is well-regarded for its industrial applications, including enzyme production and fermentation processes. *Bacillus subtilis* has also been explored for its probiotic properties, adding nutritional value beyond just protein content and quality [112]. *Rhodopseudomonas palustris*, known for its versatility in anaerobic and aerobic conditions, can reach protein contents of around 70% (dry weight w/w) [113]. This bacterium is particularly interesting for its ability to utilize a wide range of carbon sources, including waste materials, making it a candidate for sustainable protein production in bioremediation contexts.

Overall, the protein content in bacteria can vary widely, typically ranging from 40% to over 70% of the dry biomass, with the rest composed of nucleic acids, lipids, and other cellular components. This variability is influenced by factors like species, substrate type, fermentation conditions (including oxygen levels, pH, and temperature), and growth stage. An overview of the principal components of various bacterial species is compiled in Table 2.

**Table 2:** General composition of different bacteria (% of dry matter) and its applications

Source	Protein (%)	Lipid (%)	Carbohydrate (%)	Application	Reference
*Bacillus subtilis*	40–71	8	NR	Fermentation agent, protein production, probiotic	[111, 114]
*Escherichia coli*	66	8	NR		[114]
*Lactobacillus acidophilus*	68–71	NR	NR	Fish feed, high protein ingredient	[107]
*Methylococcus capsulatus*	53–81	8	NR	Fish feed, antioxidant,	[108, 115]
*Methylophilus methylotrophus*	40–70	7	NR	Animal feed	[108, 109, 116]
*Rhodocyclus gelatinosus*	68	1	28	Animal feed, biofertilizer	[117]
*Rhodopseudomonas palustris*	55–70	1–12	4–11	Animal feed, biofertilizer	[113, 118]
*Rhodopseudomonas sphaeroides*	52–67	NR	NR	Animal feed, high protein ingredient	[119, 120]

*NR* Not reported

### Fungal protein

Fungi constitute a diverse group of heterotrophic organisms that have gained significant attention for their potential in microbial protein production. Fungi's adaptability to grow on a wide array of substrates, including agricultural and industrial waste, positions them as a sustainable source of protein, aiding in waste management while providing high-quality nutrition [121, 122]. Their potential in food technology, from meat alternatives to nutritional supplements, underscores the multifaceted utility of fungal proteins in modern diets and industries.

Various fungal species are explored for their high protein content and versatile nutritional profile. *Fusarium venenatum* is perhaps the most commercially known, used in the production of mycoprotein, which forms the basis of the meat substitute Quorn [123]. This filamentous fungus can produce proteins that constitute up to 45% of its dry weight. *Fusarium venenatum* is particularly valued for its textural properties, mimicking meat, and its rich content of essential amino acids, fiber, and vitamins [124]. This fungus can be processed into various food products like burgers, nuggets, and even pasta, offering a versatile protein source. Beyond its nutritional profile, *Fusarium venenatum* has been linked to health benefits, including improved digestive health due to its high fiber content, potential cholesterol-lowering effects, and the provision of essential vitamins and minerals that contribute to overall well-being [124]. *Aspergillus oryzae*, another well-studied fungus, is utilized in both food fermentation and protein production, offering protein contents of around 40% [125]. It's renowned for its role in traditional foods like soy sauce and sake but has also been engineered to enhance its protein yield and nutritional profile [126]. *Aspergillus oryzae* is noted for its ability to produce a variety of enzymes and bioactive compounds, contributing to its health benefits. *Saccharomyces cerevisiae* (baker's or brewer's yeast) is a yeast with significant protein content, typically around 40–50% of its dry mass [127]. Known for its role in baking and brewing, yeast is also appreciated in the health sector for its B-vitamin content, minerals, and fiber, making it a valuable supplement for both humans and animals [39].

The protein content in fungi can range broadly from 30% to over 60% of dry biomass, with the remainder including carbohydrates, lipids, and fiber. This composition varies significantly with species, cultivation substrates (which can range from simple sugars to complex lignocellulosic materials), fermentation conditions (oxygen availability, pH, temperature), and growth phase. Research into the biochemical composition of fungi has led to extensive documentation in the literature. To offer an overview of their key components, data from various fungal species have been synthesized in Table 3.

**Table 3:** General composition of different fungi (% of dry matter) and its applications

Source	Protein (%)	Lipid (%)	Carbohydrate (%)	Application	Reference
*Aspergillus niger*	49–51	NR	NR	Fermentation agent, biofilm forming isolate, protein production	[107, 122, 128]
*Aspergillus oryzae*	40–48	3	44	Fermentation agent, protein production	[125, 129, 130]
*Candida utilis*	26–56	3–4	2–4	Flavoring and seasoning agent, feed ingredient	[131–133]
*Cryptococcus aureus*	52–57	NR	NR	Fish feed	[134, 135]
*Fusarium venenatum*	45	13–24	10–25	Meat substitute *Quorn*	[123, 124, 136]
*Neurospora crassa*	42–48	3–4	35–41	Meat substitute, enhance meat-based products	[137]
*Neurospora intermedia*	55	16	56	Meat substitute, enhance meat-based products	[129]
*Pleorotus ostratus*	14–42	0.5–5	37–48	Edible mushroom, nutraceutical, improvement of antioxidant ability and rheological properties in yogurts	[138–140]
*Rhizopus oligosporus*	50	NR	NR		[129]
*Saccharomyces cerevisiae*	40–48	0.5–2	31–39	Antioxidant, emulsifier, foaming agent, high protein ingredient	[127, 141]

*NR* Not reported

### Microbial protein production fermentation systems

Microbial protein production can be achieved through two primary methods: solid-state fermentation and submerged fermentation. After fermentation, the microbial biomass undergoes downstream processing for further refinement.

#### Solid-state fermentation

Solid State Fermentation involves cultivating microorganisms on solid substrates in the absence of free water, closely simulating their natural environments and facilitating efficient substrate utilization [142]. Suitable substrates include pretreated biomasses like sugarcane bagasse, rice straw, and corn stover, which provide accessible carbohydrates for microbial growth. For example, Samadi et al. [143] utilized alkali-treated bagasse with *Saccharomyces cerevisiae*, achieving a microbial protein content of 13% in the final biomass. Similarly, Khalil et al. [144] fermented pretreated rice bran and bagasse with *Pleurotus sajorcaju*, resulting in 26 g of microbial protein per kg of substrate. Lourens [145] demonstrated the efficacy of deacetylated and disk-refined bagasse fermentation with *Pleurotus ostreatus* and *Fusarium venenatum*, yielding 25 and 33 g of protein per kg of treated bagasse, respectively. However, these findings are based on laboratory-scale experiments, and the feasibility of scaling up to biorefinery levels remains to be explored.

#### Submerged fermentation

In contrast, submerged fermentation involves growing microorganisms in liquid media, which offers better control over environmental parameters, nutrient availability, and process scalability. This method can utilize sugar-rich enzymatic hydrolysates for microbial protein production. Zhao et al. [146] demonstrated the use of bagasse hydrolysate with *Candida utilis*, which resulted in 132 g of microbial protein per kg of bagasse, with 56% of it being crude protein. In another study by Sun et al. [147] used *Trichoderma cutaneum* to ferment sugars from the enzymatic hydrolysis of ammonia-treated brewer's spent grain, achieving 310 g of protein per kg of substrate. However, the use of sugar hydrolysates for microbial protein competes with ethanol production. Alternatives include lower-value biorefining streams like pretreatment liquor or vinasse from distillation. Pretreatment liquor, rich in acetate and lignin, is obtained after lignocellulosic biomass pretreatment, while vinasse, an acidic slurry with high organic content, comes from the distillation process. If not managed properly, both can pose environmental risks. Nair and Taherzadeh [148] used *Neurospora intermedia* and *Aspergillus oryzae* to ferment vinasse, producing 202 and 223 g of fungal biomass with approximately 40–45% microbial protein. Nitayavardhana et al. [149] also fermented vinasse in an airlift bioreactor with *Rhizopus oligosporus*, yielding 8 g of fungal biomass per g of initial biomass. The advanced control in submerged fermentation can potentially lead to higher-quality microbial products that are suitable for applications in monogastric and fish feeds or even human consumption. Table 4 presents an overview of different microorganisms and substrates used in submerged and solid-state fermentation for microbial protein production.

**Table 4:** Examples of microorganisms and substrates for microbial protein production in submerged and solid-state fermentation

Production method	Microorganism	Substrate	Reference
Submerged fermentation	*Bacillus subtilis*	Ram horns	[114]
	*Candida tropicalis*	Sugarcane bagasse	[150]
	*Candida utilis*	Pineapple cannery effluent	[151]
	*Cryptococcus aureus*	Jerusalem artichoke extract	[134]
	*Escherichia coli*	Ram horns	[114]
	*Fusarium venenatum*	Glucose	[152]
	*Fusarium venenatum*	Date waste	[153]
	*Rhodopseudomonas sp*	Municipal wastewater	[154]
	*Rhodopseudomonas gelatinosa*	Wheat bran infusion	[155]
	*Saccharomyces cerevisiae*	Potato, orange, carrot and apple peels	[156]
Solid-state fermentation	*Aspergillus niger*	Rapeseed cake	[157]
	*Aspergillus orizae*	Black-eyed pea seed flour	[158]
	*Candida utilis*	Wheat bran	[159]
	*Fusarium venenatum*	Sugarcane bagasse	[145]
	*Neurospora intermedia*	Waste bread	[160]
	*Rhizopus oligosporus*	Wheat bran	[159]
	*Rhizopus oligosporus*	Soybean meal	[161]
	*Pleorotus ostratus*	Sugarcane bagasse	[145]
	*Pleorotus ostratus*	Canola meal	[162]
	*Saccharomyces cerevisiae*	Sugarcane bagasse	[145]

## Use of agro-industrial by-products as macro and micronutrients for fermentation

Many efforts have been made in order to advance the use of agro-industrial by-products for anaerobic and aerobic submerged fermentations. This section presents and discusses the knowledge on the use of agro-industrial by-products as sources of carbon, nitrogen and micronutrients for fermentation for the production of bioethanol and other chemical reagents.

### Carbon sources

Fermentation processes traditionally use readily available, food-grade, or easily fermentable carbon sources as macronutrients. These carbon sources typically do not require extensive pretreatment compared to by-products or agricultural waste. They include simple sugars or refined carbohydrates that microorganisms can directly metabolize. Conversely, carbon sources derived from by-products and agricultural waste offer a sustainable alternative to conventional substrates, reducing costs and environmental impact. However, these materials require pretreatment to break down complex structures into fermentable sugars and eliminate inhibitors that could hinder microbial fermentation efficiency.

Lignocellulosic biomass, encompassing agricultural residues such as corn stover, wheat, rice and soybean straw, and sugarcane bagasse, constitutes a significant carbon source due to its widespread availability and economic viability. These residues contain approximately 50–100 g/L of total sugar, predominantly glucose and xylose, following pretreatment. Pretreatment methods include physical, chemical or biochemical/enzymatic dismantling the cellulose, hemicellulose, and lignin matrix into fermentable sugars. Nonetheless, acid hydrolysis, while effective, produces inhibitors like furfural and hydroxymethylfurfural (HMF), requiring detoxification via overliming or activated carbon treatment [163].

Currently, many efforts have been made to utilize agricultural by-products to produce bioethanol to be used mainly as fuel. *Saccharomyces cerevisiae* engineered for xylose metabolism utilizes hydrolysates with 60 g per liter of glucose and 30 g per liter of xylose to produce ethanol at high yields [164]. Molasses, a by-product of sugar refining from sugarcane or beets, have been used due to its cost-effectiveness. However, its high impurities require intensive downstream purification. For ethanol production, *Saccharomyces cerevisiae* ferments pretreated molasses at 200 g per liter of sucrose producing 70 to 100 g per liter of ethanol in 48 to 72 h [165]. Similarly, *Aspergillus niger* employs molasses at 140 to 200 g per liter for citric acid production, achieving yields exceeding 100 g per liter after 5 to 7 days under submerged fermentation [166]. Cheese whey, a co-product from cheese manufacturing, contains lactose that can be fermented by lactic acid bacteria to lactic acid. Its high-water content and presence of proteins and salts require ultrafiltration to concentrate lactose to 100 to 150 g per liter and remove proteins, or enzymatic hydrolysis with β-galactosidase to produce glucose and galactose for organisms lacking lactose metabolism. *Kluyveromyces marxianus* fermented pretreated whey at 100 g per liter of lactose, generating 40 to 50 g per liter of ethanol, with yields optimized by pH and temperature control [167]. In addition, *Propionibacterium* species utilize whey lactose at 30 to 50 g per liter for propionic acid production, achieving 15 to 20 g per liter following pretreatment [168].

### Nitrogen sources

Nitrogen sources are critical in fermentation processes, providing the essential building blocks for microbial growth, protein synthesis and metabolic activities. While conventional sources like ammonium salts and urea are widely used, by-products and agricultural wastes offer sustainable alternatives. However, these alternatives require pretreatments to make nitrogen bioavailable or to remove inhibitors. Several studies have successfully utilized nitrogen-rich by-products and wastes in fermentation following appropriate pretreatments.

Corn steep liquor (CSL), a by-product of corn wet-milling, is rich in organic nitrogen (20 to 50 g per liter total nitrogen, including amino acids, peptides and proteins). Its complex composition, including high solids and salts, necessitates pretreatments to remove insoluble residue and sometimes dilution to 10 to 30% (v/v) to adjust nitrogen levels (5 to 15 g per liter) and reduce viscosity. In *Clostridium acetobutylicum* acetone-butanol-ethanol fermentation, CSL at 10 to 20 g per liter served as a cost-effective substitute for yeast extract by enhancing solvent production (20 to 25 g per liter of solvents) [169]. Similarly, *Lactobacillus* species utilizes CSL at 5 to 10 g per liter for lactic acid production, achieving comparable yields to chemically defined media after optimized pH control [170]. Soybean meal, a by-product of soy oil extraction, contains 40 to 50% (w/w) protein (70 to 80 g per liter of nitrogen on a dry basis) and serves as a low-cost nitrogen source in fermentation. Acid or enzymatic hydrolysis breaks down protein into peptides and amino acids improving bioavailability. Neutralization or filtration removes residual solids and inhibitors like phytic acid. *Bacillus subtilis* ferments soybean meal hydrolysate at 15 to 20 g per liter of nitrogen for protease production, with enzyme yields reaching 200 to 300 U/mL [171]. *Saccharomyces cerevisiae* uses soybean meal hydrolysate at 10 to 15 g per liter of nitrogen for ethanol production, with yields comparable to defined media [165]. Fish waste, including offal and trimmings from seafood processing contains 10 to 15% of protein (20 to 30 g per liter of nitrogen), which can be hydrolyzed with enzymes or acids to yield peptides and amino acids with 5 to 15 g per liter of soluble nitrogen*. Propionibacterium freudenreichii* and *Candida utilis* ferments fish waste hydrolysate at 10 g per liter of nitrogen to yield 15 to 20 g per liter of propionic acid and 20 to 30 g per liter biomass respectively [168, 172]. Distillers’ Dried Grains with Solubles (DDGS), a by-product from corn ethanol production from grains, provides 25 to 35% protein (40 to 69 g per liter of nitrogen). Pretreatment involves enzymatic hydrolysis with proteases or acid treatment to solubilize proteins into peptides and amino acids (about 5 to 15 g per liter nitrogen) after processing. The pretreatment is concluded with filtration or centrifugation to remove insoluble fiber. *Aspergillus niger* fermented DDGS hydrolysate at 10 g per liter nitrogen for citric acid production, achieving 80 to 100 g per liter with optimized process conditions [166]. Similarly, *Saccharomyces cerevisiae* utilized DDGS hydrolysate at 5 to 10 g per liter of nitrogen for bioethanol fermentation, reaching 30 to 40 g per liter [173]. Other nitrogen-rich agro-industrial wastes like poultry feather meal, rice bran, and soy molasses have also been successfully valorized via microbial fermentation through appropriate pretreatment and optimization [166, 171]. When comparing nitrogen and carbon sources, the current scientific knowledge will greatly benefit from more fundamental and applied research on the utilization of alternative nitrogen sources, considering the economic and environmental costs associated with standard nitrogen sources.

### Micronutrients

Micronutrients, including vitamins and minerals, are vital in fermentation processes by acting as cofactors, catalysts, or structural components that enhance microbial metabolism, growth, and product formation. Although needed in trace amounts, these nutrients significantly influence fermentation efficiency and yield [174, 175]. In addition to conventional sources, upcycled materials provide sustainable alternatives for supplying these micronutrients, thereby reducing costs and environmental impact.

Magnesium is a critical mineral in fermentation, acting as a cofactor for enzymes such as hexokinase and phosphofructokinase in glycolysis, and stabilizing ATP. Concentrations typically range from 0.1 to 5 mM in media, depending on the organism and process. In ethanol production by *Saccharomyces cerevisiae*, magnesium at 0.5 to 2 mM enhanced yeast growth and ethanol yields, often reaching 50 to 60 g per liter from 150 g per liter glucose. Magnesium can be sourced from spent grains, a by-product that contains 1 to 2 mg magnesium/g spent grain in dry weight [176, 177]. Zinc (Zn2⁺) is essential for fermentation, serving as a cofactor for alcohol dehydrogenase in ethanol production and supporting protein synthesis. Typical concentrations range from 0.01 to 0.5 mM. For *Saccharomyces cerevisiae*, zinc at 0.1 to 0.2 mM improves fermentation efficiency, elevating ethanol concentrations by 10 to 15%. Seafood processing waste can be upcycled as a source, as it contains zinc concentrations of 200 µg/g dry weight [178, 179]. Iron (Fe2⁺/Fe3⁺) plays a role in fermentation as a component of cytochromes and iron-sulfur proteins in the electron transport chain, supporting respiration and redox balance. Typical requirements range from 0.001 to 0.1 mM due to its low solubility and potential toxicity at higher levels. In citric acid production by *Aspergillus niger*, iron at 0.01 to 0.05 mM optimizes yields, reaching 100 to 120 g per liter from 200 g per liter of sucrose. Iron can be sourced from molasses, a sugar refining by-product, containing 0.1 to 1 mg/g iron [166]. Vitamin B Complex, including biotin (B7) and thiamine (B1), supports fermentation by aiding coenzyme functions. Biotin, at 0.001 to 0.01 mg/L, enhances lipid synthesis and carboxylase activity in *Propionibacterium* species, boosting propionic acid yields to 15 to 20 g per liter from 40 g per liter glycerol [168]. Thiamine, at 0.1 to 1 mg per liter, improves glycolysis in *Saccharomyces cerevisiae*, increasing ethanol production by 5 to 10%. Brewer’s yeast sludge, a brewery waste, could be upcycled as a source of vitamin B complex as it is rich in B vitamins (0.5 to 2 mg/g dry weight) [180]. Lastly, manganese (Mn2⁺) acts as a cofactor for enzymes like superoxide dismutase, protecting microbes from oxidative stress, and is used at 0.01 to 0.5 mM. In *Lactobacillus* lactic acid fermentation, manganese at 0.05 to 0.2 mM enhances cell viability and yields, achieving 80 to 90 g per liter lactic acid from 100 g per liter glucose. Manganese is present in fruit and vegetable peels, providing a renewable source for fermentation. However, alkaline extraction or composting pretreatments need to be done to increase manganese’s bioavailability [170].

Upcycled products like spent grains, seafood wastes, molasses, brewer’s yeast sludge and fruit/vegetable peels show promise as sustainable sources of micronutrients in fermentation, requiring moderately complex pretreatments like composting, extraction or hydrolysis to ensure the micronutrient’s bioavailability. Leveraging those residues has the potential to not only reduce fermentation costs but also to reduce the environmental impact of both the disposal of waste as well as the production of synthetic forms of micronutrients.

The overall number of studies evaluating alternative sources of micronutrients, when compared to carbon or even nitrogen sources, is limited. In this context, the utilization of agro-industrial by-products as sources of micronutrients for fermentation will significantly benefit from more studies on evaluating new and current sources, processing techniques to improve bioavailability, as well as a better understanding of the economic and environmental impacts associated with it.

## Strategies for the upcycling of agro-industrial by-products for microbial protein production

The utilization of agricultural and agro-industrial by-products for microbial protein production represents a promising and sustainable strategy for reducing waste while simultaneously addressing global protein demands. By-products generated from agricultural and processing activities are typically rich in carbohydrates, proteins, or other essential nutrients that can serve as substrates for fungal fermentation. Many of these by-products fall into the category of lignocellulosic residues, which include leafy or woody biomass that are abundant, widely available, and underutilized. These residues are primarily composed of cellulose, hemicellulose, and lignin, which require pretreatment to release fermentable sugars for fungal growth. Another key group of by-products includes fruit waste—such as peels, cores, and seeds from oranges, bananas, and apples—which are rich in carbohydrates, vitamins, and minerals, providing nutrient-dense media for microbial fermentation. Key examples of agro-industrial by-products include the following:

*Sugarcane bagasse*

This fibrous residue is generated after sugar extraction from sugarcane stalks. It is a lignocellulosic material rich in cellulose and hemicellulose, making it an excellent substrate for fungal fermentation. However, its sugars are not readily bioavailable, and pretreatment techniques, such as alkaline deacetylation or steam explosion, are often required to break down its structure [145].

*Corn stover*

Comprising the leaves, stalks, and cobs left after corn harvesting, corn stover is another lignocellulosic residue with high cellulose and hemicellulose content. Pretreatment is essential to solubilize its sugars for fungal metabolism, as its lignin fraction can limit accessibility [181].

*Wheat straw*

A by-product of wheat harvesting, wheat straw consists of lignin, cellulose, and hemicellulose. Its widespread availability and carbohydrate composition make it a valuable feedstock for fungal fermentation processes, though its structural complexity also necessitates pretreatment [182].

*Rice straw*

Generated after rice harvesting, rice straw is a lignocellulosic material similar to wheat straw but often more abundant in rice-producing regions. It has significant potential as a fungal substrate following appropriate pretreatment [183].

*Soybean hulls*

These outer shells are a by-product of soybean processing and are rich in fiber, proteins, and carbohydrates. They are a promising resource for fungal protein production with suitable nutrient adjustments [184].

*Cotton stalks*

These are the woody residues left after cotton harvesting. Cotton stalks are lignocellulosic materials rich in cellulose and hemicellulose, making them suitable substrates for fungal fermentation. Their abundance in cotton-producing regions and the potential for valorization through pretreatment processes highlight their importance as a sustainable resource for mycoprotein production [185].

*Almond hulls*

These fibrous by-products are generated during almond processing and consist of the outer layers of the almond fruit. Almond hulls are rich in carbohydrates, fiber, and some residual sugars, making them a viable substrate for fungal fermentation [186].

*Rice bran*

A by-product of rice milling, rice bran is nutritionally dense, containing proteins, fats, and carbohydrates. Its minimal processing requirements make it highly suitable for fungal fermentation, offering a cost-effective and sustainable substrate [187].

*Wheat bran*

Produced during flour milling, wheat bran is composed of starch, protein, and dietary fiber. Its composition supports fungal growth, especially when supplemented with additional nutrients [159].

*Brewer's spent grain*

This by-product of beer brewing consists of residual grains left after malt extraction. Rich in proteins and fibers, Brewer's spent grain provides a nutrient-dense medium for fungal fermentation, with significant potential for upcycling into mycoprotein [188, 189].

*Molasses*

A viscous, sugar-rich residue from sugar refining processes, molasses is an inexpensive and readily available carbohydrate source. It is widely utilized in microbial fermentations, including those for mycoprotein production, due to its affordability and high sugar content [190, 191].

*Fruit waste*

This includes residues such as peels, cores, and seeds generated during juice or food processing. Examples include orange peels, banana peels, and apple cores, which are rich in carbohydrates, dietary fiber, and vitamins, providing excellent substrates for fungal growth [192, 193].

While lignocellulosic residues offer considerable potential for microbial protein production, their complex structure often limits direct utilization. Pretreatment techniques are necessary to break down cellulose and hemicellulose into fermentable sugars. These methods also help solubilize lignin, which, while recalcitrant and non-fermentable, can sometimes be utilized as an additional carbon source under specific conditions [194]. Effective pretreatment strategies thus play a critical role in enhancing substrate bioavailability and ensuring efficient fungal fermentation for mycoprotein production.

### Pretreatments for lignocellulosic biomass prior to microbial fermentation

The utilization of lignocellulosic biomass for mycoprotein production requires overcoming its natural recalcitrance to microbial degradation. Lignocellulose is composed of a complex matrix of cellulose, hemicellulose, and lignin, where cellulose and hemicellulose serve as carbon sources for fungal growth [195]. However, the rigid structure, reinforced by lignin, restricts the bioavailability of fermentable sugars. To enhance the accessibility of these sugars, various pretreatment techniques are employed. Pretreatments aim to disrupt the lignocellulosic structure, remove lignin or hemicellulose, and increase the surface area for enzymatic hydrolysis or microbial activity [196]. These methods can generally be categorized into physical, chemical, physicochemical, and biological. The choice of pretreatment depends on the substrate characteristics, desired sugar yields, process economics, and environmental impact.

### Physical pretreatments

Physical pretreatments involve mechanical and physical processes to reduce particle size, increase the surface area, and alter the physical structure of lignocellulosic biomass. The primary objective is to increase the accessibility of cellulose and hemicellulose by reducing crystallinity and improving porosity. Common physical pretreatment methods include:

#### *Milling and Grinding*

These processes involve reducing the particle size of biomass using equipment such as hammer mills, ball mills, or knife mills. By reducing the biomass to smaller particle sizes, milling increases the surface area available for enzymatic attack and microbial growth. However, the energy demand for fine milling can be high, and combining this approach with other pretreatments, such as deacetylation, can help reduce energy costs [197].

#### *Chipping and extrusion*

Chipping reduces the biomass into larger, coarse pieces, making it more manageable for subsequent pretreatment steps. Extrusion, on the other hand, applies heat, pressure, and shear forces to disrupt the lignocellulosic structure, reducing crystallinity and improving digestibility [198].

#### *Ultrasound-assisted pretreatment*

Ultrasound treatment utilizes high-frequency sound waves (20–100 kHz) to generate cavitation bubbles in a liquid medium. These bubbles collapse, releasing localized high energy that causes shear forces and disrupts the biomass structure. Ultrasound-assisted pretreatment can enhance enzyme accessibility, reduce cellulose crystallinity, and increase surface area. Additionally, it has the advantage of being a non-thermal method, making it particularly suitable for heat-sensitive biomass components [199].

#### *Cold plasma*

Plasma is the fourth state of matter which is composed of high energy and it can be used in several fields such as semiconductors, food processing, medical sterilization, waste degradation and enhancing substrate utilization and biomass yields. Recent studies have used cold plasma to detoxify sugar cane hydrolyzed biomass enhancing its applicability as a carbon source for microbial fermentation and, in combination with Fe^2+^, was used to depolymerize the cellulose structure in pineapple peel [200, 201].

### Chemical pretreatments

Chemical pretreatments are designed to alter the chemical composition of lignocellulosic biomass, either by removing lignin, solubilizing hemicellulose, or reducing the crystallinity of cellulose. These pretreatments improve the release of fermentable sugars, making them highly effective for fungal fermentation processes.

#### *Alkaline pretreatment*

Alkaline pretreatment involves using alkaline agents such as sodium hydroxide (NaOH), potassium hydroxide (KOH), or aqueous ammonia to remove lignin and disrupt the lignocellulosic matrix. This process increases the porosity of the biomass and improves cellulose accessibility. Sugarcane bagasse and corn stover, for instance, are commonly pretreated with NaOH to enhance sugar yields. Alkaline treatments are particularly advantageous due to their ability to minimize sugar degradation and the formation of inhibitory compounds [202, 203].

#### *Acid pretreatment*

Acid pretreatment uses diluted acids, such as sulfuric acid (H₂SO₄) or hydrochloric acid (HCl), to hydrolyze hemicellulose and release fermentable sugars. This method effectively solubilizes hemicellulose into monomeric sugars, but it can generate inhibitory compounds such as hydroxymethylfurfural (HMF) and acetic acid, which may hinder microbial fermentation. As such, detoxification steps may be necessary following acid pretreatment [204, 205].

#### *Organosolv pretreatment*

Organosolv pretreatment involves the use of organic solvents, such as ethanol or acetone, often in combination with acidic or alkaline catalysts. This method selectively removes lignin while preserving cellulose and hemicellulose, making the biomass more amenable to enzymatic hydrolysis. Additionally, lignin recovered from organosolv pretreatment can be valorized for other applications, enhancing the economic feasibility of the process [206].

#### *Oxidative pretreatment*

Oxidative pretreatment employs oxidizing agents such as hydrogen peroxide (H₂O₂) or ozone to break down lignin and reduce its inhibitory effects on microbial growth. For example, hydrogen peroxide pretreatment of almond hulls and rice straw has demonstrated significant improvement in sugar release and fungal fermentation efficiency [207].

#### *Ammonia fiber expansion (AFEX)*

AFEX pretreatment uses liquid ammonia to swell the lignocellulosic structure and break the hydrogen bonds within cellulose. This process enhances the digestibility of cellulose and hemicellulose while preserving their structural integrity. AFEX is particularly effective for agricultural residues such as corn stover and wheat straw [208–210].

#### Physicochemical pretreatments

Physicochemical pretreatments combine physical and chemical processes to enhance biomass deconstruction. These methods are well-suited for large-scale operations due to their effectiveness and reduced chemical inputs compared to purely chemical methods. Two of the most widely used methods are:

#### *Steam explosion*

This method involves exposing biomass to high-pressure steam followed by rapid depressurization. The sudden pressure drop disrupts the biomass structure, solubilizing hemicellulose and reducing lignin content. Steam explosion is an energy-efficient approach and is particularly effective for materials like sugarcane bagasse, corn stover, and wheat straw [211].

#### *Liquid hot water (LHW)*

LHW pretreatment involves treating biomass with hot water (160–230 °C) under pressure. The heat and pressure promote hemicellulose solubilization without requiring chemicals, reducing the risk of inhibitor formation [212, 213].

### Biological pretreatments

Biological pretreatments utilize microorganism's enzymes, primarily fungi, to degrade lignin and hemicellulose selectively. White-rot fungi (e.g., *Phanerochaete chrysosporium* or *Trametes versicolor*) produce ligninolytic enzymes, such as lignin peroxidase, manganese peroxidase, and laccase, which target lignin while preserving cellulose for hydrolysis. Biological pretreatments are environmentally friendly, require mild operating conditions, and produce fewer inhibitors. However, they are slower compared to physical or chemical methods, often requiring weeks to achieve significant biomass degradation. Despite their longer processing times, biological pretreatments can be integrated with other methods to reduce energy and chemical inputs while maintaining high efficiency [214].

## Life cycle assessment of the use of agro-industrial by-products on fermentation-based processes

Life Cycle Assessment (LCA) is a standardized methodology, as outlined in ISO 14040 and 14,044, used to evaluate the environmental impacts of products or processes across their entire life cycle, from raw material extraction to end-of-life disposal [215]. LCA can provide valuable insights into the sustainability of using upcycled by-products and by-products as potential feedstocks for fermentation, cultivated meat, and microbial protein [216–218]. The goal of this section is to present and discuss the current literature on LCA of fermentation-based processes using upcycled feedstocks, detailing their environmental impacts, key findings, and potential benefits.

Fermentation processes often leverage upcycled by-products to produce value-added products like biofuels, organic acids, and enzymes. However, not enough studies have been done on fermented products using upcycled feedstock. One study assessed the environmental impacts of cultivated meat production compared to conventional meat [75]. While the study primarily used conventional feedstocks, it provides a baseline GWP of 4.7 kg CO₂-eq per kg of cultivated meat, significantly lower than beef (27 kg CO₂-eq per kg). The study suggests that using upcycled feedstocks, such as brewer’s spent grain or whey, could further reduce impacts, aligning with the potential for lower GWP and land use seen in other upcycled processes. Another research used LCA to compare the environmental impacts of producing microbial protein ovalbumin using *Trichoderma reesei* culture and suggested that low-carbon energy diminishes the environmental impact of chicken-egg-derived ovalbumin [219]. The microbial process could potentially utilize upcycled feedstocks like lignocellulosic hydrolysates. The research noted industrial processes associated with glucose consumption as an environmental hot spot, highlighting the environmental advantage of microbial processes with upcycled inputs [75, 219]. These LCA studies consistently demonstrate that fermentation, cultivated meat and microbial protein production processes leveraging upcycled by-products and waste streams can significantly reduce environmental impacts, with GWP reductions ranging from 20 to 90% when compared to conventional methods. However, challenges include energy-intensive pretreatments (15 to 60% of impacts) and scalability, suggesting areas for further research to optimize processes and reduce energy use [220].

## Mycoproteins, a case study of microbial protein commercial success

Among the microbial proteins commercially available or used for the production of meat-like products, mycoproteins from various sources have been particularly successful. They have established economically viable production systems, penetrated the alternative protein market, and gained significant consumer acceptance [221].

Quorn/Monde Nissin Corporation is the most well-known producer of mycoprotein, using *Fusarium venenatum* biomass as the main ingredient in their Quorn products since 1970 [222]. Another company that utilizes *Fusarium venenatum* is ENOUGH (3fbio Ltd) producing ABUNDA mycoprotein which operates alongside a Cargil starch facility that provides fermentable sugars, decreasing the environmental impact of the mycoprotein production, showcasing a successful case study of the application of new technologies to improve the sustainability of alternative proteins [223]. Another company Nature’s Fynd in Chicago, Illinois, for example, uses *Fusarium flavolapis*, a fungus found originally in Yellowstone Park in Wyoming, to make meatless patties and a cream-cheese alternative [224].

Mycoprotein, produced from the filamentous fungus *Fusarium venenatum*, was first developed in the 1960 s when concerns over global food security led to efforts to identify alternative protein sources that could be produced efficiently [152]. Initial research by British scientists, who focused on fungal fermentation to produce protein-rich biomass, eventually led to the commercial product known today as Quorn, which was introduced in the UK in the 1980 s. Advancements in fermentation technology have driven the evolution of mycoprotein. Unlike traditional livestock protein, which requires extensive resources, mycoprotein is produced through continuous fermentation in controlled conditions, significantly reducing land, water, and energy use and offering a more environmentally efficient alternative [124]. Consumer acceptance has been essential to the evolution of mycoprotein. Initially, its market was limited to the UK,however, as global demand for sustainable proteins has increased, Quorn and other mycoprotein-based products have expanded into markets across the United States, Europe, and Asia. Today, mycoprotein is among the leading microbial protein sources, appealing to vegetarians, flexitarians, and individuals concerned with the environmental impact of conventional meat production, with its health and nutritional benefits being primary factors in consumer choice [225]. Further research is needed focusing on optimizing mycoprotein production to improve texture, flavor, and nutritional properties. Additionally, developments in genetic and metabolic engineering may allow enhanced strains with higher nutrient density and digestibility. However, challenges remain, including reducing production costs to make mycoprotein more accessible and addressing regulatory issues in new markets. *Fusarium venetatum* is known to be mechanical labile and the shear applied by stirred tanks during submerged fermentation may pose some challenges. In this sense, it is commonly produced using airlift bioreactors [226]. However, there are still challenges when working towards bioprocessing scale-up, due to low oxygenation during the descending phase in the bioreactor which may decrease production yield or cause fungal mutation [226, 227].

Other than *Fusarium venenatum*, *Neurospora crassa* is another fungal strain of commercial importance in the mycoprotein market [137]. Meati Foods and The Better Meat Co., both based in the United States of America, utilize *Neurospora crassa* to produce their mycoprotein products and ingredients [228–230]. One advantage of *Neurospora crassa* is its biomass's superior resistance to mechanical stress compared to *Fusarium venenatum* [231]. This allows for the use of stirred tank bioreactors, which are cheaper, readily available, and better understood in terms of general operation and parameter optimization [232, 233]. The journey of *Neurospora crassa* as a mycoprotein source for meat alternatives can be traced back to its natural occurrence and historical use in food. *Neurospora crassa*, commonly known as red bread mold, has been part of human dietary practices, particularly in Southeast Asia, where it is used to produce “oncom”, a fermented food like tempeh. Oncom is made from by-products like peanuts press cake or okara (soybean residue), showcasing *Neurospora crassa*’s ability to convert agricultural waste into nutritious food [234]. *Neurospora crassa* first gained scientific attention not for its food potential but for its utility in genetic research. Its life cycle, ease of cultivation and well-characterized genetics made it a model organism for genetic studies beginning in the mid-twentieth century. The work of George Beadle and Edward Tatum on *Neurosopora crassa* in the 1940 s, which earned them a Nobel Prize in the 1940 s, highlighted its role in understanding gene function, laying the groundwork for its later exploration in food science [235].

The shift toward considering *Neurospora crassa* as a mycoprotein for food applications came with increased interest in sustainable protein sources amid growing concerns about the environmental impact of traditional meat production. Unlike *Fusarium venenatum*, the fungus behind the well-known mycoprotein Quorn, *Neurospora crassa* has a broader historical use in food, which might ease regulatory approval and consumer acceptance. Early studies focused on the safety and nutritional profile of *Neurospora crassa* mycoprotein. Research confirmed its long history of safe use in Asian cuisines, high protein content (up to 45% in dry weight), essential amino acid composition comparable to animal proteins, and the presence of beneficial nutrients like dietary fiber, vitamins and minerals [137]. Furthermore, the application of biotechnological techniques such as submerged fermentation and solid-state fermentation, has been pivotal in scaling up production from *Neurospora crassa*. These methods allow for high yields of biomass with controlled nutritional content, effectively adapting traditional fermentation processes to modern industrial standards.

Additional information on startups and established companies in the field of fungal biomass for mycoprotein for commercial application is presented in Table 5.

**Table 5:** Companies commercializing and/or developing mycoprotein products and ingredients

Company name	Fungal Strain	Country	Reference
Quorn/Monde Nissin Corporation	*Fusarium venenatum*	United Kingdom	[222]
ENOUGH	*Fusarium venenatum*	The Netherlands	[223]
Nature’s Fynd	*Fusarium flavolapis*	United States of America	[224]
Meati Foods	Neurosopora crassa	United States of America	[229]
The Better Meat Co	Neurosopora crassa	United States of America	[228]
Enifer	*Paecilomyces variotii*	Finland	[236, 237]
MyForest Foods	*Pleurotus ostreatus*	United State of America	[238]
Mycorena	Not disclosed	Sweeden	[239]
Mycovation	Not disclosed	Singapore	[240]
Aqua Cultured Foods	Not disclosed	United States of America	[241]
KIDEMIS GmbH	Not disclosed	Switzerland	[242]
Funki	Not disclosed	Estonia	[243]
Libre Foods	Not disclosed	Spain	[244]
Tempty Foods	Not disclosed	Denmark	[245]

**Fig. 1:**
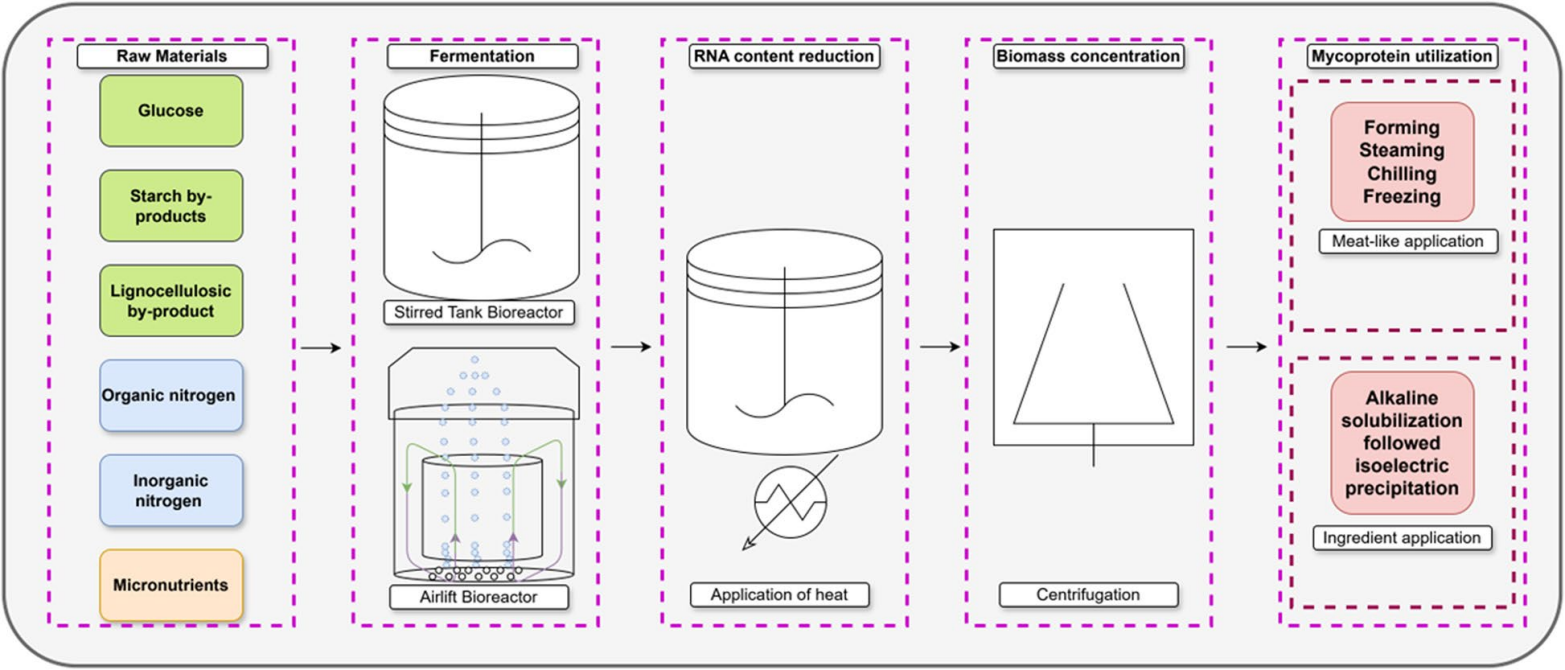
Example of unit operations for the production of mycoprotein for alternative meat-like products or protein extraction to be used as an ingredient

Once the fungal biomass is produced one important step to ensure that the mycoprotein is safe for human consumption is the reduction of the RNA content to below 2% (w/w on dry mass) [246, 247]. This is achieved by subjecting the fungal biomass to thermal treatment of 72 to 75 °C for 30 to 45 min [12, 227]. Since this is a step that demands high thermal energy for a long period of time, investigating alternatives to optimize this step without sacrificing the safety and quality of the final product is needed [248]. There is great potential for the use of emerging technologies as possible alternatives to reduce RNA content in a more efficient time and energy manner [249–251]. After reducing the RNA content, the biomass is concentrated by centrifugation to 20% to 25% in solids, and then it goes through a series of steaming, chilling and freezing operations to texturize the mycoprotein into a meat-like product or it can undergo protein extraction to be used as an ingredient in product formulation (Fig. 1) [12, 248, 252].

## Limitations and opportunities for microbial protein production

Many of the technical limitations observed for cultivated meats are also true for microbial proteins. One of the foremost challenges in microbial protein production is the transition from laboratory scale to industrial scale. While small-scale experiments can yield promising results, scaling up these processes involves significant hurdles. Issues like maintaining consistent conditions across larger volumes, managing heat and mass transfer problems, and ensuring uniform nutrient distribution become critical [253]. Similarly, with cultivated meat production, the complexity of bioreactor design for mycoprotein increases with scale, requiring sophisticated control systems to manage variables like pH, oxygen levels, and temperature. Additionally, the economic viability of scaling up must be considered, as the costs associated with larger reactors, energy, and maintenance can be prohibitive [38]. Efficient bioprocess engineering and optimization are essential to make microbial protein production commercially feasible on a large scale.

Contamination is another significant challenge in both solid-state and submerged fermentation systems. Microbial cultures are susceptible to contamination by undesirable bacteria, fungi and viruses, which can compromise the quality and safety of the protein product [109]. In solid-state fermentation, the solid substrates can harbor unwanted microbes, making sterilization or sanitation of the substrate a complex task. In submerged fermentation, despite better control, contamination can still occur if there are failures in sterility maintenance of the media or equipment. Ensuring purity involves rigorous monitoring employing sterile techniques, which can add to production costs and raise concerns about residues in the final product. The challenge is to achieve high yields of microbial protein while maintaining a sterile environment to guarantee product safety and quality.

The acceptance of microbial proteins as a food source among consumers is a considerable cultural and may be a psychological barrier. Despite the nutritional benefits, there's a general wariness towards foods produced through microbial processes, often associated with unfamiliarity or misconceptions about fermentation and biotechnology [254]. Public education on the safety, benefits, and sustainability of microbial proteins is necessary to change perceptions. Regulatory approval for human consumption can also be a lengthy process, requiring extensive safety assessments and consumer studies to ensure the product meets health standards and public expectations. Overcoming this challenge involves not only scientific validation but also effective communication strategies to demystify microbial proteins and highlight their role in sustainable food systems.

Even when microbial proteins are accepted, ensuring they meet nutritional standards and sensory expectations is challenging. Microbial proteins might have high nucleic acid content, which can be a health concern if consumed in large amounts, necessitating additional processing steps to reduce them [255]. The taste, texture, and overall sensory profile of microbial proteins often differ from traditional protein sources, potentially limiting their use in food applications unless modified. Techniques like 3D printing, texturization, or blending with other ingredients are used to improve acceptability, but these processes can complicate production and increase costs [256]. Furthermore, the nutritional profile, including the balance of amino acids, vitamins, and minerals, needs to be tailored or supplemented to match or exceed those of conventional proteins, which requires additional research and development in bioprocessing and formulation.

Each of these challenges requires a multifaceted approach involving biotechnology, engineering, regulatory, and consumer education to further advance microbial protein production and acceptance as a sustainable and integral part of the global food supply chain.

### Integration of microbial protein production with the bioenergy sector

**Fig. 2:**
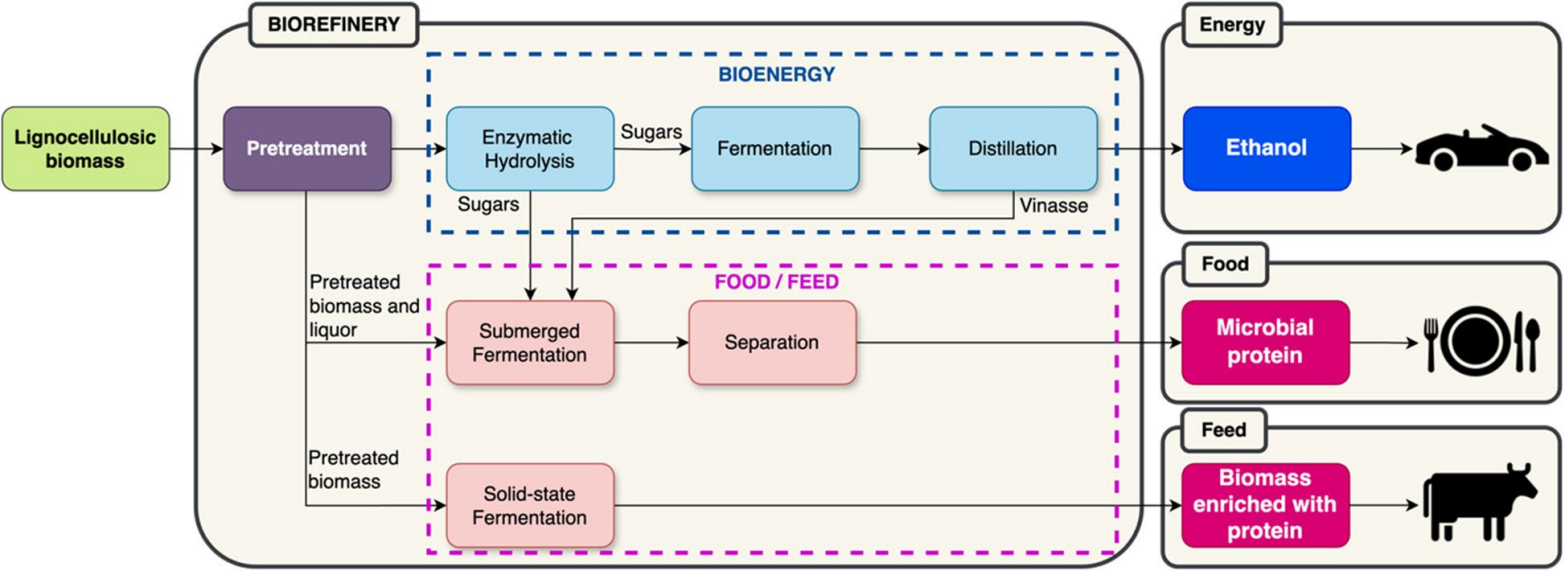
Synergist integration between energy, food and animal feed production

The synergy between microbial protein production and the bioenergy sector offers a multifaceted approach to addressing both energy and protein needs sustainably. Starting with lignocellulosic biomass, a prevalent raw material in bioenergy production, the process begins with pretreatment to break down the complex structure of the biomass, making its components more accessible for further conversion [202, 257, 258]. This pretreated biomass can be directed towards two primary pathways: one for biofuel production and another for protein-rich feed and food (Fig. 2).

In the biofuel pathway, the pretreated biomass undergoes enzymatic hydrolysis to convert cellulose and hemicellulose into fermentable sugars, followed by fermentation to produce ethanol. The distillation process then separates ethanol from the fermentation broth, yielding ethanol as the final product alongside vinasse, a nutrient-rich byproduct. This biofuel route not only attends the energy sector but also generates byproducts that can be repurposed for protein production.

Parallel to this, the second pathway integrates the food sector by using the pretreated biomass, hydrolysate from enzymatic hydrolysis, and vinasse from distillation as substrates for submerged fermentation. This method allows for the cultivation of microorganisms in liquid media, where they can convert these substrates into microbial proteins suitable for human consumption. The proteins produced can be tailored for specific nutritional profiles or functional properties, becoming ingredients in food products ranging from protein supplements to meat and dairy alternatives.

The third pathway integrates the feed sector by using the pretreated biomass in solid-state fermentation. Here, the biomass supports the growth of protein-producing microorganisms, resulting in lignocellulosic biomass infused with high-quality microbial protein. This product can directly serve as a nutritional feed for livestock, offering an alternative to conventional protein sources like soy or fishmeal, which are often linked with significant environmental impacts.

This approach not only valorizes waste streams from biofuel production but also contributes to sustainable food and feed production by reducing reliance on traditional agricultural practices that consume vast amounts of land and water. By integrating microbial protein production within the bioenergy sector, a closed-loop system can be created where waste from one process becomes the feedstock for another, thus enhancing the overall efficiency, sustainability, and economic viability of both energy and food production systems.

## Conclusions

Microbial proteins and cultivated meat have the potential to advance sustainable protein production, addressing both demand and nutritional needs, as well as environmental concerns. The successful commercialization of mycoproteins demonstrates the viability of microbial proteins as a mainstream food source. These proteins offer significant advantages, including high nutritional value, efficient production processes, and reduced environmental impact compared to traditional animal-based proteins.

The use of agricultural and industrial by-products as substrates for microbial protein production is a promising strategy to enhance sustainability. By upcycling waste materials like sugarcane bagasse, corn stover, and fruit peels, the environmental footprint of protein production can be reduced while simultaneously addressing waste management issues. This approach not only provides a cost-effective feedstock for microbial fermentation but also contributes to a circular economy, where waste is transformed into valuable resources.

Furthermore, the integration of microbial protein production with the bioenergy sector offers a synergistic solution to global energy and food challenges. Utilizing by-products from biofuel production as substrates for microbial fermentation moves current bioenergy production systems towards a closed-loop system that maximizes resource efficiency and minimizes waste. This integration has the potential to significantly reduce the costs and environmental impact of both protein and biofuel production.

The potential of using agricultural by-products in the growth media for cultivated meat is another relevant avenue for reducing costs and environmental impacts. Cultivated meat, produced through cellular agriculture, requires nutrient-rich media for cell growth. By incorporating agricultural by-products into these media has the potential to lower production costs and enhance the sustainability of cultivated meat, making it a more viable alternative to conventional meat.

In conclusion, microbial proteins, particularly mycoproteins in the current state along with cultivated meat, are at the forefront of sustainable protein innovation for meat-like products. Their ability to utilize diverse substrates, including agricultural by-products, positions them as key components in the transition towards a more sustainable and resilient food system. Continued research and development in this field will be crucial to overcoming current challenges and unlocking the full potential of alternative proteins in addressing global food security and environmental sustainability.

## Data Availability

No datasets were generated or analysed during the current study.

## References

[1] Berners-Lee, M., Kennelly, C., Watson, R., & Hewitt, C. N. (2018). Current global food production is sufficient to meet human nutritional needs in 2050 provided there is radical societal adaptation. Elementa, 6. 10.1525/ELEMENTA.310/112838

[2] Wu G. Dietary protein intake and human health. Food Funct. 2016;7(3):1251–65. 10.1039/C5FO01530H. 26797090 10.1039/c5fo01530h

[3] Smith NW, Fletcher AJ, Hill JP, McNabb WC. Modeling the Contribution of Meat to Global Nutrient Availability. Front Nutr. 2022;9: 766796. 10.3389/FNUT.2022.766796/BIBTEX. 35187029 10.3389/fnut.2022.766796 PMC8849209

[4] Le Mouël C, Forslund A. How can we feed the world in 2050? A review of the responses from global scenario studies. Eur Rev Agric Econ. 2017;44(4):541–91. 10.1093/ERAE/JBX006.

[5] Röös, E., Sundberg, C., Tidåker, P., Strid, I., & Hansson, P. A. (2013). Can carbon footprint serve as an indicator of the environmental impact of meat production? Ecological Indicators, 24. 10.1016/j.ecolind.2012.08.004

[6] FAO. (2024). Meat Market Review. Emerging trends and outlook in 2024.

[7] FAO. (2022). Meat Market Review. Overview of market and policy developments 2021.

[8] van der Laan S, Breeman G, Scherer L. Animal Lives Affected by Meat Consumption Trends in the G20 Countries. Animals. 2024;14(11):1662. 10.3390/ANI14111662/S1. 38891709 10.3390/ani14111662 PMC11171019

[9] Spiro A, Hill Z, Stanner S. Meat and the future of sustainable diets—Challenges and opportunities. Nutr Bull. 2024;49(4):572–98. 10.1111/NBU.12713. 39526859 10.1111/nbu.12713

[10] Risner D, McDonald KA, Jones C, Spang ES. A techno-economic model of mycoprotein production: achieving price parity with beef protein. Frontiers in Sustainable Food Systems. 2023;7:1204307. 10.3389/FSUFS.2023.1204307/BIBTEX.

[11] Upcraft T, Tu WC, Johnson R, Finnigan T, Van Hung N, Hallett J, Guo M. Protein from renewable resources: mycoprotein production from agricultural residues. Green Chem. 2021;23(14):5150–65. 10.1039/D1GC01021B.

[12] Ahmad, M. I., Farooq, S., Alhamoud, Y., Li, C., & Zhang, H. (2022). A review on mycoprotein: History, nutritional composition, production methods, and health benefits. In Trends in Food Science and Technology (Vol. 121, pp. 14–29). Elsevier Ltd. 10.1016/j.tifs.2022.01.027

[13] Uzundumlu, A. S., & Dilli, M. (2023). Estimating Chicken Meat Productions of Leader Countries for 2019–2025 Years. Ciencia Rural, 53(2). 10.1590/0103-8478CR20210477

[14] Gerber PJ, Hristov AN, Henderson B, Makkar H, Oh J, Lee C, Meinen R, Montes F, Ott T, Firkins J, Rotz A, Dell C, Adesogan AT, Yang WZ, Tricarico JM, Kebreab E, Waghorn G, Dijkstra J, Oosting S. Technical options for the mitigation of direct methane and nitrous oxide emissions from livestock: a review. In Animal : an international journal of animal bioscience: 2013;7(Suppl):2. 10.1017/S1751731113000876. 10.1017/S1751731113000876 23739465

[15] Holmes, C. D., Prather, M. J., Søvde, O. A., & Myhre, G. (2013). Future methane, hydroxyl, and their uncertainties: Key climate and emission parameters for future predictions. Atmospheric Chemistry and Physics, 13(1). 10.5194/acp-13-285-2013

[16] Eshel, G., Shepon, A., Makov, T., & Milo, R. (2015). Partitioning United States’ feed consumption among livestock categories for improved environmental cost assessments. Journal of Agricultural Science, 153(3). 10.1017/S0021859614000690

[17] Strassburg BBN, Latawiec AE, Barioni LG, Nobre CA, da Silva VP, Valentim JF, Vianna M, Assad ED. When enough should be enough: Improving the use of current agricultural lands could meet production demands and spare natural habitats in Brazil. Glob Environ Chang. 2014;28(1):84–97. 10.1016/J.GLOENVCHA.2014.06.001.

[18] de Vries M, de Boer IJM. Comparing environmental impacts for livestock products: A review of life cycle assessments. Livest Sci. 2010;128(1–3):1–11. 10.1016/J.LIVSCI.2009.11.007.

[19] Summary, E., Alig, M., Grandl, F., Mieleitner, J., Nemecek, T., Gaillard, G., Basset-Mens, C., Van Der Werf, H. M. G., Castellini, C., Boggia, A., Cortina, C., Dal Bosco, A., Paolotti, L., Novelli, E., Mugnai, C., Dourmad, J. Y., Ryschawy, J., Trousson, T., Bonneau, M., … Bébin, D. (2012). Evaluation of the livestock sector’s contribution to the EU greenhouse gas emissions (GGELS)–final report. Journal of Cleaner Production, 105(4).

[20] Walker, P., Rhubart-Berg, P., McKenzie, S., Kelling, K., & Lawrence, R. S. (2005). Public health implications of meat production and consumption. Public Health Nutrition, 8(4). 10.1079/phn2005727 10.1079/phn2005727 15975179

[21] Grossmann, L., & Weiss, J. (2021). Alternative Protein Sources as Technofunctional Food Ingredients. Annual Review of Food Science and Technology, 12. 10.1146/annurev-food-062520-093642 10.1146/annurev-food-062520-093642 33472014

[22] Pojić, M., Mišan, A., & Tiwari, B. (2018). Eco-innovative technologies for extraction of proteins for human consumption from renewable protein sources of plant origin. In Trends in Food Science and Technology (Vol. 75, pp. 93–104). Elsevier Ltd. 10.1016/j.tifs.2018.03.010

[23] Jeske S, Zannini E, Arendt EK. Past, present and future: The strength of plant-based dairy substitutes based on gluten-free raw materials. Food Res Int. 2018;110:42–51. 10.1016/J.FOODRES.2017.03.045. 30029705 10.1016/j.foodres.2017.03.045

[24] Mellor, C., Embling, R., Neilson, L., Randall, T., Wakeham, C., Lee, M. D., & Wilkinson, L. L. (2022). Consumer Knowledge and Acceptance of “Algae” as a Protein Alternative: A UK-Based Qualitative Study. Foods 2022, Vol. 11, Page 1703, 11(12), 1703. 10.3390/FOODS11121703 10.3390/foods11121703 PMC9223121 35741901

[25] Grossmann, L., Hinrichs, J., & Weiss, J. (2020). Cultivation and downstream processing of microalgae and cyanobacteria to generate protein-based technofunctional food ingredients. In Critical Reviews in Food Science and Nutrition (Vol. 60, Issue 17). 10.1080/10408398.2019.1672137 10.1080/10408398.2019.1672137 31595777

[26] Schwenzfeier, A., Helbig, A., Wierenga, P. A., & Gruppen, H. (2013). Emulsion properties of algae soluble protein isolate from Tetraselmis sp. Food Hydrocolloids, 30(1). 10.1016/j.foodhyd.2012.06.002

[27] Grossmann, L., Ebert, S., Hinrichs, J., & Weiss, J. (2019). Formation and Stability of Emulsions Prepared with a Water-Soluble Extract from the Microalga Chlorella protothecoides. Journal of Agricultural and Food Chemistry, 67(23). 10.1021/acs.jafc.8b05337 10.1021/acs.jafc.8b05337 31099556

[28] Ebert, S., Grossmann, L., Hinrichs, J., & Weiss, J. (2019). Emulsifying properties of water-soluble proteins extracted from the microalgae: Chlorella sorokiniana and Phaeodactylum tricornutum. Food and Function, 10(2). 10.1039/c8fo02197j 10.1039/c8fo02197j 30667441

[29] Good Food Institute. (2021). State of the Industry Report | Cultivated Meat.

[30] Kirsch, M., Morales-Dalmau, J., & Lavrentieva, A. (2023). Cultivated meat manufacturing: Technology, trends, and challenges. In Engineering in Life Sciences (Vol. 23, Issue 12). 10.1002/elsc.202300227 10.1002/elsc.202300227 PMC10711323 38089567

[31] Congressional Research Service. (2023). Cell-cultivated Meat: an Overview. Biotechnology and Bioengineering, 118(8).

[32] Martínez, E. A., dos Santos, J. F., Araujo, G. S., de Souza, S. M. A., de Cássia Lacerda Brambilla Rodrigu, R., & Canettieri, E. V. (2018). Production of Single Cell Protein (SCP) from Vinasse (pp. 215–238). 10.1007/978-3-319-90379-8_10

[33] Bajić, B., Vučurović, D., Vasić, Đ., Jevtić-Mučibabić, R., & Dodić, S. (2023). Biotechnological Production of Sustainable Microbial Proteins from Agro-Industrial Residues and By-Products. In Foods (Vol. 12, Issue 1). MDPI. 10.3390/foods12010107 10.3390/foods12010107 PMC9818480 36613323

[34] Zepka LQ, Jacob-Lopes E, Goldbeck R, Souza-Soares LA, Queiroz MI. Nutritional evaluation of single-cell protein produced by Aphanothece microscopica Nägeli. Biores Technol. 2010;101(18):7107–11. 10.1016/j.biortech.2010.04.001. 10.1016/j.biortech.2010.04.001 20417094

[35] Tian, Y., Li, J., Meng, J., & Li, J. (2023). High-yield production of single-cell protein from starch processing wastewater using co-cultivation of yeasts. Bioresource Technology, 370, 128527. 10.1016/j.biortech.2022.128527 10.1016/j.biortech.2022.128527 36572157

[36] Acosta, N., Sakarika, M., Kerckhof, F.-M., Law, C. K. Y., De Vrieze, J., & Rabaey, K. (2020). Microbial protein production from methane via electrochemical biogas upgrading. Chemical Engineering Journal, 391, 123625. 10.1016/j.cej.2019.123625

[37] Yang, X., Xu, M., Zou, R., Angelidaki, I., & Zhang, Y. (2021). Microbial protein production from CO2, H2, and recycled nitrogen: Focusing on ammonia toxicity and nitrogen sources. Journal of Cleaner Production, 291, 125921. 10.1016/j.jclepro.2021.125921

[38] Ugbogu, E. A., & Ugbogu, O. C. (2016). a Review of Microbial Protein Production: Prospects and Challenges. FUW Trends in Science & Technology Journal, 1(1).

[39] Schmitz LM, Kreitli N, Obermaier L, Weber N, Rychlik M, Angenent LT. Power-to-vitamins: producing folate (vitamin B_9_) from renewable electric power and CO_2_ with a microbial protein system. Trends Biotechnol. 2024;42(12):1691–714. 10.1016/j.tibtech.2024.06.014. 39271416 10.1016/j.tibtech.2024.06.014

[40] Sharif, M., Zafar, M. H., Aqib, A. I., Saeed, M., Farag, M. R., & Alagawany, M. (2021). Single cell protein: Sources, mechanism of production, nutritional value and its uses in aquaculture nutrition. Aquaculture, 531, 735885. 10.1016/j.aquaculture.2020.735885

[41] Koukoumaki, D. I., Tsouko, E., Papanikolaou, S., Ioannou, Z., Diamantopoulou, P., & Sarris, D. (2024). Recent advances in the production of single cell protein from renewable resources and applications. In Carbon Resources Conversion (Vol. 7, Issue 2). KeAi Publishing Communications Ltd. 10.1016/j.crcon.2023.07.004

[42] Hertzler, S. R., Lieblein-Boff, J. C., Weiler, M., & Allgeier, C. (2020). Plant proteins: Assessing their nutritional quality and effects on health and physical function. In Nutrients (Vol. 12, Issue 12, pp. 1–27). MDPI AG. 10.3390/nu12123704 10.3390/nu12123704 PMC7760812 33266120

[43] Zhou, H., Hu, Y., Tan, Y., Zhang, Z., & McClements, D. J. (2021). Digestibility and gastrointestinal fate of meat versus plant-based meat analogs: An in vitro comparison. Food Chemistry, 364. 10.1016/j.foodchem.2021.130439 10.1016/j.foodchem.2021.130439 34186477

[44] Reynaud, Y., Buffière, C., Cohade, B., Vauris, M., Liebermann, K., Hafnaoui, N., Lopez, M., Souchon, I., Dupont, D., & Rémond, D. (2021). True ileal amino acid digestibility and digestible indispensable amino acid scores (DIAASs) of plant-based protein foods. Food Chemistry, 338. 10.1016/J.FOODCHEM.2020.128020 10.1016/j.foodchem.2020.128020 32932087

[45] Kerry Health And Nutrition Institute. (2020). Ten Key Health and Nutrition Trends 2020. Kerry Health And Nutrition Institute.

[46] Estell, M., Hughes, J., & Grafenauer, S. (2021). Plant protein and plant-based meat alternatives: Consumer and nutrition professional attitudes and perceptions. Sustainability (Switzerland), 13(3). 10.3390/su13031478

[47] Christ, J., & Sprinkle, D. (2020). U.S. Food Market Outlook 2020: Home Cooking, Grocery Shopping, & Food Trends in the Age of Coronavirus. Packaged Facts. https://www.packagedfacts.com/Food-Outlook-Home-Cooking-Grocery-Shopping-Trends-Age-Coronavirus-13331998/

[48] Scharf, A., Kasel, U., Wichmann, G., & Besler, M. (2013). Performance of ELISA and PCR methods for the determination of allergens in food: An evaluation of six years of proficiency testing for soy (Glycine max L.) and wheat gluten (Triticum aestivum L.). Journal of Agricultural and Food Chemistry, 61(43). 10.1021/jf402619d 10.1021/jf402619d 24144233

[49] Henchion, M., Hayes, M., Mullen, A. M., Fenelon, M., & Tiwari, B. (2017). Future protein supply and demand: Strategies and factors influencing a sustainable equilibrium. Foods, 6(7). 10.3390/foods6070053 10.3390/foods6070053 PMC5532560 28726744

[50] Aschemann-Witzel J, Gantriis RF, Fraga P, Perez-Cueto FJA. Plant-based food and protein trend from a business perspective: markets, consumers, and the challenges and opportunities in the future. In Critical Reviews in Food Science and Nutrition. 2020. 10.1080/10408398.2020.1793730. 10.1080/10408398.2020.1793730 32654499

[51] Formanski, K. (2019). Plant-based Proteins - US - May 2019. Mintel. https://reports.mintel.com/display/919520/?fromSearch=%3Ffreetext%3Dchickpea%2520protein

[52] Srivastava, N. (2020). Patent insights: next-gen plant protein ingredients. Mintel. https://clients.mintel.com/report/patent-insights-next-gen-plant-protein-ingredients?fromSearch=%3Ffreetext%3Doat%2520protein

[53] Wansink, B. (2003). How Do Front and Back Package Labels Influence Beliefs about Health Claims? In Journal of Consumer Affairs (Vol. 37, Issue 2). 10.1111/j.1745-6606.2003.tb00455.x

[54] McCarthy, K. S., Parker, M., Ameerally, A., Drake, S. L., & Drake, M. A. (2017). Drivers of choice for fluid milk versus plant-based alternatives: What are consumer perceptions of fluid milk? Journal of Dairy Science, 100(8). 10.3168/jds.2016-12519 10.3168/jds.2016-12519 28551193

[55] Marinangeli, C. P. F., Mansilla, W. D., & Shoveller, A. K. (2018). Navigating protein claim regulations in North America for foods containing plant-based proteins. In Cereal Foods World (Vol. 63, Issue 5). 10.1094/CFW-63-5-0207

[56] Boukid, F. (2021). Plant-based meat analogues: from niche to mainstream. In European Food Research and Technology (Vol. 247, Issue 2). 10.1007/s00217-020-03630-9

[57] Boye, J. I., Aksay, S., Roufik, S., Ribéreau, S., Mondor, M., Farnworth, E., & Rajamohamed, S. H. (2010). Comparison of the functional properties of pea, chickpea and lentil protein concentrates processed using ultrafiltration and isoelectric precipitation techniques. Food Research International, 43(2). 10.1016/j.foodres.2009.07.021

[58] Dempsey, C., & Bryant, C. J. (2020). Cultured meat: Do Chinese consumers have an appetite? 10.31219/osf.io/pjm83

[59] Whiting, K. (2020). How soon will we be eating lab grown meat?” . https://Www.Weforum.Org/Stories/2020/10/Will-We-Eat-Lab-Grown-Meat-World-Food-Day/.

[60] Post, M. J. (2012). Cultured meat from stem cells: Challenges and prospects. In Meat Science (Vol. 92, Issue 3, pp. 297–301). 10.1016/j.meatsci.2012.04.008 10.1016/j.meatsci.2012.04.008 22543115

[61] Edelman, P. D., McFarland, D. C., Mironov, V. A., & Matheny, J. G. (2005). Commentary: In Vitro -Cultured Meat Production. Tissue Engineering, 11(5–6), 659–662. 10.1089/ten.2005.11.659 10.1089/ten.2005.11.659 15998207

[62] Benjaminson, M. A., Gilchriest, J. A., & Lorenz, M. (2002). IN VITRO EDIBLE MUSCLE PROTEIN PRODUCTION SYSTEM (MPPS): STAGE 1, FISH. In Acta Astronautica (Vol. 51, Issue 12). www.elsevier.com/locate/actaastro 10.1016/s0094-5765(02)00033-4 12416526

[63] Post, M. J. (2014). An alternative animal protein source: Cultured beef. Annals of the New York Academy of Sciences, 1328(1). 10.1111/nyas.12569 10.1111/nyas.12569 25376889

[64] Kim, C. J., Kim, S. H., Lee, E. Y., Son, Y. M., Bakhsh, A., Hwang, Y. H., & Joo, S. T. (2023). Optimal temperature for culturing chicken satellite cells to enhance production yield and umami intensity of cultured meat. Food Chemistry Advances, 2. 10.1016/j.focha.2023.100307

[65] Ryu, M., Kim, M., Jung, H. Y., Kim, C. H., & Jo, C. (2023). Effect of p38 inhibitor on the proliferation of chicken muscle stem cells and differentiation into muscle and fat. Animal Bioscience, 36(2). 10.5713/ab.22.0171 10.5713/ab.22.0171 PMC9834727 36108703

[66] Verbruggen, S., Luining, D., van Essen, A., & Post, M. J. (2018). Bovine myoblast cell production in a microcarriers-based system. Cytotechnology, 70(2). 10.1007/s10616-017-0101-8 10.1007/s10616-017-0101-8 PMC5851947 28470539

[67] Xu H, Wan H, Zuo W, Sun W, Owens RT, Harper JR, Ayares DL, McQuillan DJ. A porcine-derived acellular dermal scaffold that supports soft tissue regeneration: Removal of terminal galactose-α-(1,3)-galactose and retention of matrix structure. Tissue Engineering - Part A. 2009;15(7):1807–19. 10.1089/ten.tea.2008.0384. 19196142 10.1089/ten.tea.2008.0384

[68] Rubio, N., Datar, I., Stachura, D., Kaplan, D., & Krueger, K. (2019). Cell-Based Fish: A Novel Approach to Seafood Production and an Opportunity for Cellular Agriculture. In Frontiers in Sustainable Food Systems (Vol. 3). Frontiers Media S.A. 10.3389/fsufs.2019.00043

[69] Gabillard, J. C., Sabin, N., & Paboeuf, G. (2010). In vitro characterization of proliferation and differentiation of trout satellite cells. Cell and Tissue Research, 342(3). 10.1007/s00441-010-1071-8 10.1007/s00441-010-1071-8 21086139

[70] Zhang F, Zhu F, Yang FX, Hao JP, Hou ZC. Genomic selection for meat quality traits in Pekin duck. Anim Genet. 2022;53(1):94–100. 10.1111/age.13157. 34841553 10.1111/age.13157

[71] GOOD Meat. (2025, February 17). GOOD Meat at the butchery. https://Www.Goodmeat.Co/.

[72] Bakhsh, A., Lee, E. Y., Ncho, C. M., Kim, C. J., Son, Y. M., Hwang, Y. H., & Joo, S. T. (2022). Quality Characteristics of Meat Analogs through the Incorporation of Textured Vegetable Protein: A Systematic Review. In Foods (Vol. 11, Issue 9). 10.3390/foods11091242 10.3390/foods11091242 PMC9100116 35563965

[73] Ben-Arye, T., Shandalov, Y., Ben-Shaul, S., Landau, S., Zagury, Y., Ianovici, I., Lavon, N., & Levenberg, S. (2020). Textured soy protein scaffolds enable the generation of three-dimensional bovine skeletal muscle tissue for cell-based meat. Nature Food, 1(4). 10.1038/s43016-020-0046-5

[74] Letti, L. A. J., Karp, S. G., Molento, C. F. M., Colonia, B. S. O., Boschero, R. A., Soccol, V. T., Herrmann, L. W., Penha, R. de O., Woiciechowski, A. L., & Soccol, C. R. (2021). Cultivated meat: recent technological developments, current market and future challenges. Biotechnology Research and Innovation, 5(1). 10.4322/biori.202101

[75] Tuomisto, H. L., & Teixeira De Mattos, M. J. (2011). Environmental impacts of cultured meat production. Environmental Science and Technology, 45(14). 10.1021/es200130u 10.1021/es200130u 21682287

[76] Stephens, N., Di Silvio, L., Dunsford, I., Ellis, M., Glencross, A., & Sexton, A. (2018). Bringing cultured meat to market: Technical, socio-political, and regulatory challenges in cellular agriculture. In Trends in Food Science and Technology (Vol. 78, pp. 155–166). Elsevier Ltd. 10.1016/j.tifs.2018.04.010 10.1016/j.tifs.2018.04.010 PMC6078906 30100674

[77] United States Department of Agriculture. (2023). Human Food Made with Cultured Animal Cells.

[78] MarketsandMarkets. (2024). Cultured Meat Industry - Forthcoming Trends to Fuel the Global Growth. https://Www.Marketsandmarkets.Com/ResearchInsight/Emerging-Trends-in-Cultured-Meat-Market.Asp.

[79] Future Market Insights. (2022). Cultured Meat Market Insights – Lab-Grown Innovation & Market Expansion 2023 to 2033.

[80] Grand View Research. (2023). Cultured Meat Market Size, Share & Trends Report Cultured Meat Market Size, Share & Trends Analysis Report By Source (Poultry, Beef, Seafood, Pork, Duck), By End-use (Nuggets, Burgers, Meatballs, Sausages, Hot Dogs), By Region, And Segment Forecasts, 2023 - 2030.

[81] Shah-Neville, W. (2024). 6 cultured meat companies making waves. https://Www.Labiotech.Eu/Best-Biotech/Cultured-Meat-Companies/.

[82] Bryant, C. J., & Barnett, J. C. (2019). What’s in a name? Consumer perceptions of in vitro meat under different names. Appetite, 137. 10.1016/j.appet.2019.02.021 10.1016/j.appet.2019.02.021 30840874

[83] Bryant, C., & Dillard, C. (2019). The impact of framing on acceptance of cultured meat. Frontiers in Nutrition, 6. 10.3389/fnut.2019.00103 10.3389/fnut.2019.00103 PMC6616100 31334244

[84] Siegrist, M., & Hartmann, C. (2020). Consumer acceptance of novel food technologies. In Nature Food (Vol. 1, Issue 6). 10.1038/s43016-020-0094-x 10.1038/s43016-020-0094-x 37128090

[85] Broad GM. Making meat, better: the metaphors of plant-based and cell-based meat innovation. Environ Commun. 2020;14(7):919–32. 10.1080/17524032.2020.1725085.

[86] Mattick, C. S. (2018). Cellular agriculture: The coming revolution in food production. Bulletin of the Atomic Scientists, 74(1). 10.1080/00963402.2017.1413059

[87] Singapore Food Agency. (2021). GROWING our FOOD FUTURE.

[88] Kulus, M., Jankowski, M., Kranc, W., Golkar Narenji, A., Farzaneh, M., Dzięgiel, P., Zabel, M., Antosik, P., Bukowska, D., Mozdziak, P., & Kempisty, B. (2023). Bioreactors, scaffolds and microcarriers and in vitro meat production—current obstacles and potential solutions. In Frontiers in Nutrition (Vol. 10). Frontiers Media SA. 10.3389/fnut.2023.1225233 10.3389/fnut.2023.1225233 PMC10513094 37743926

[89] Marcellin, E., Bansal, N., Ebert, B., Gumulya, Y., Johnson, H., Peng, H., Turner, M., & van der Pols, J. (2024). Precision Fermentation: A Future of Food in Australia.

[90] Specht, E. A., Welch, D. R., Rees Clayton, E. M., & Lagally, C. D. (2018). Opportunities for applying biomedical production and manufacturing methods to the development of the clean meat industry. Biochemical Engineering Journal, 132. 10.1016/j.bej.2018.01.015

[91] Moutsatsou, P., Ochs, J., Schmitt, R. H., Hewitt, C. J., & Hanga, M. P. (2019). Automation in cell and gene therapy manufacturing: from past to future. In Biotechnology Letters (Vol. 41, Issue 11, pp. 1245–1253). Springer Netherlands. 10.1007/s10529-019-02732-z 10.1007/s10529-019-02732-z PMC6811377 31541330

[92] Barkallah M, Dammak M, Louati I, Hentati F, Hadrich B, Mechichi T, Ayadi MA, Fendri I, Attia H, Abdelkafi S. Effect of Spirulina platensis fortification on physicochemical, textural, antioxidant and sensory properties of yogurt during fermentation and storage. LWT. 2017;84:323–30. 10.1016/j.lwt.2017.05.071.

[93] Batista AP, Niccolai A, Fradinho P, Fragoso S, Bursic I, Rodolfi L, Biondi N, Tredici MR, Sousa I, Raymundo A. Microalgae biomass as an alternative ingredient in cookies: Sensory, physical and chemical properties, antioxidant activity and in vitro digestibility. Algal Res. 2017;26:161–71. 10.1016/j.algal.2017.07.017.

[94] Zanella, L., & Vianello, F. (2020). Microalgae of the genus Nannochloropsis: Chemical composition and functional implications for human nutrition. In Journal of Functional Foods (Vol. 68). Elsevier Ltd. 10.1016/j.jff.2020.103919

[95] Acateca-Hernández, M. I., Hernández-Cázares, A. S., Hidalgo-Contreras, J. V., Jiménez-Munguía, M. T., & Ríos-Corripio, Ma. A. (2024). Evaluation of the functional properties of a protein isolate from Arthrospira maxima and its application in a meat sausage. Heliyon, 10(13), e33500. 10.1016/j.heliyon.2024.e33500 10.1016/j.heliyon.2024.e33500 PMC11255854 39027591

[96] Spolaore P, Joannis-Cassan C, Duran E, Isambert A. Commercial applications of microalgae. J Biosci Bioeng. 2006;101(2):87–96. 10.1263/jbb.101.87. 16569602 10.1263/jbb.101.87

[97] Becker, E. W. (2007). Micro-algae as a source of protein. Biotechnology Advances, 25(2), 207–210. 10.1016/j.biotechadv.2006.11.002 10.1016/j.biotechadv.2006.11.002 17196357

[98] Safi, C., Zebib, B., Merah, O., Pontalier, P. Y., & Vaca-Garcia, C. (2014). Morphology, composition, production, processing and applications of Chlorella vulgaris: A review. In Renewable and Sustainable Energy Reviews (Vol. 35, pp. 265–278). Elsevier Ltd. 10.1016/j.rser.2014.04.007

[99] Matos ÂP, Feller R, Moecke EHS, de Oliveira JV, Junior AF, Derner RB, Sant’Anna, E. S. Chemical Characterization of Six Microalgae with Potential Utility for Food Application. JAOCS, Journal of the American Oil Chemists’ Society. 2016;93(7):963–72. 10.1007/s11746-016-2849-y.

[100] Tibbetts SM, Bjornsson WJ, McGinn PJ. Biochemical composition and amino acid profiles of Nannochloropsis granulata algal biomass before and after supercritical fluid CO2 extraction at two processing temperatures. Anim Feed Sci Technol. 2015;204:62–71. 10.1016/j.anifeedsci.2015.04.006.

[101] Letsiou, S., Kalliampakou, K., Gardikis, K., Mantecon, L., Infante, C., Chatzikonstantinou, M., Labrou, N. E., & Flemetakis, E. (2017). Skin protective effects of Nannochloropsis gaditana extract on H2O2-stressed human dermal fibroblasts. Frontiers in Marine Science, 4(JUL). 10.3389/fmars.2017.00221

[102] Menegol T, Romero-Villegas GI, López-Rodríguez M, Navarro-López E, López-Rosales L, Chisti Y, Cerón-García MC, Molina-Grima E. Mixotrophic production of polyunsaturated fatty acids and carotenoids by the microalga Nannochloropsis gaditana. J Appl Phycol. 2019;31(5):2823–32. 10.1007/s10811-019-01828-3.

[103] Castro-Ferreira, C., Gomes-Dias, J. S., Ferreira-Santos, P., Pereira, R. N., Vicente, A. A., & Rocha, C. M. R. (2022). Phaeodactylum tricornutum extracts as structuring agents for food applications: Physicochemical and functional properties. Food Hydrocolloids, 124, 107276. 10.1016/j.foodhyd.2021.107276

[104] da Silva, M. E. T., Leal, M. A., de Oliveira Resende, M., Martins, M. A., & dos Reis Coimbra, J. S. (2021). Scenedesmus obliquus protein concentrate: A sustainable alternative emulsifier for the food industry. Algal Research, 59, 102468. 10.1016/j.algal.2021.102468

[105] Fito J, Alemu K. Microalgae–bacteria consortium treatment technology for municipal wastewater management. Nanotechnology for Environmental Engineering. 2018;4(1):4. 10.1007/s41204-018-0050-2.

[106] LaTurner, Z. W., Bennett, G. N., San, K.-Y., & Stadler, L. B. (2020). Single cell protein production from food waste using purple non-sulfur bacteria shows economically viable protein products have higher environmental impacts. Journal of Cleaner Production, 276, 123114. 10.1016/j.jclepro.2020.123114

[107] Kam S, Kenari AA, Younesi H. Production of Single Cell Protein in Stickwater by Lactobacillus acidophilus and Aspergillus niger. J Aquat Food Prod Technol. 2012;21(5):403–17. 10.1080/10498850.2011.605539.

[108] Zha, X., Tsapekos, P., Zhu, X., Khoshnevisan, B., Lu, X., & Angelidaki, I. (2021). Bioconversion of wastewater to single cell protein by methanotrophic bacteria. Bioresource Technology, 320, 124351. 10.1016/j.biortech.2020.124351 10.1016/j.biortech.2020.124351 33161316

[109] Ritala, A., Häkkinen, S. T., Toivari, M., & Wiebe, M. G. (2017). Single cell protein-state-of-the-art, industrial landscape and patents 2001–2016. In Frontiers in Microbiology (Vol. 8, Issue OCT). Frontiers Media S.A. 10.3389/fmicb.2017.02009 10.3389/fmicb.2017.02009 PMC5645522 29081772

[110] Kuźniar, A., Furtak, K., Włodarczyk, K., Stępniewska, Z., & Wolińska, A. (2019). Methanotrophic bacterial biomass as potential mineral feed ingredients for animals. International Journal of Environmental Research and Public Health, 16(15). 10.3390/ijerph16152674 10.3390/ijerph16152674 PMC6696423 31357395

[111] Berzina, I., Kalnins, M., Geiba, Z., Raita, S., Palcevska, J., Mika, T., & Spalvins, K. (2024). Creating Single‐Cell Protein‐Producing Bacillus subtilis Mutants Using Chemical Mutagen and Amino Acid Inhibitors. Scientifica, 2024(1). 10.1155/sci5/8968295 10.1155/sci5/8968295 PMC11623996 39649941

[112] Lefevre, M., Racedo, S. M., Denayrolles, M., Ripert, G., Desfougères, T., Lobach, A. R., Simon, R., Pélerin, F., Jüsten, P., & Urdaci, M. C. (2017). Safety assessment of Bacillus subtilis CU1 for use as a probiotic in humans. Regulatory Toxicology and Pharmacology, 83, 54–65. 10.1016/j.yrtph.2016.11.010 10.1016/j.yrtph.2016.11.010 27825987

[113] Kim, J. K., & Lee, B.-K. (2000). Mass production of Rhodopseudomonas palustris as diet for aquaculture. Aquacultural Engineering, 23(4), 281–293. 10.1016/S0144-8609(00)00057-1

[114] Kurbanoglu, E. B., & Algur, O. F. (2002). Single-cell protein production from ram horn hydrolysate by bacteria. Bioresource Technology, 85(2), 125–129. 10.1016/S0960-8524(02)00094-9 10.1016/s0960-8524(02)00094-9 12227535

[115] Yu, H., Liang, H., Longshaw, M., Wang, J., Ge, X., Ren, M., & Zhang, L. (2022). Methanotroph (Methylococcus capsulatus, Bath) bacteria meal (FeedKind®) could effectively improve the growth, apparent digestibility coefficient, blood biochemical parameters, antioxidant indices of juvenile Jian carp (Cyprinus carpio var. Jian). Animal Feed Science and Technology, 288, 115293. 10.1016/j.anifeedsci.2022.115293

[116] Øverland M, Tauson A-H, Shearer K, Skrede A. Evaluation of methane-utilising bacteria products as feed ingredients for monogastric animals. Arch Anim Nutr. 2010;64(3):171–89. 10.1080/17450391003691534. 20578647 10.1080/17450391003691534

[117] Helena E, Ponsano G, Lacava PM, Pinto MF. Chemical Composition of Rhodocyclus gelatinosus Biomass Produced in Poultry Slaughterhouse Wastewater. Braz Arch Biol Technol. 2003;46(2):143–7.

[118] Kornochalert N, Kantachote D, Chaiprapat S, Techkarnjanaruk S. Use of Rhodopseudomonas palustris P1 stimulated growth by fermented pineapple extract to treat latex rubber sheet wastewater to obtain single cell protein. Annals of Microbiology. 2014;64(3):1021–32. 10.1007/s13213-013-0739-1.

[119] Noparatnaraporn N, Nagai S. Selection of rhodobacter sphaeroides p47 as a useful source of single cell protein. J Gen Appl Microbiol. 1986;32(4):351–9. 10.2323/jgam.32.351.

[120] He J, Zhang G, Lu H. Treatment of soybean wastewater by a wild strain Rhodobacter sphaeroides and to produce protein under natural conditions. Front Environ Sci Eng China. 2010;4(3):334–9. 10.1007/s11783-010-0239-5.

[121] Sar, T., Marchlewicz, A., Harirchi, S., Mantzouridou, F. T., Hosoglu, M. I., Akbas, M. Y., Hellwig, C., & Taherzadeh, M. J. (2024). Resource recovery and treatment of wastewaters using filamentous fungi. Science of The Total Environment, 951, 175752. 10.1016/j.scitotenv.2024.175752 10.1016/j.scitotenv.2024.175752 39182768

[122] Dalbanjan, N. P., Eelager, M. P., & Narasagoudr, S. S. (2024). Microbial protein sources: A comprehensive review on the potential usage of fungi and cyanobacteria in sustainable food systems. Food and Humanity, 3, 100366. 10.1016/j.foohum.2024.100366

[123] Strong, P. J., Self, R., Allikian, K., Szewczyk, E., Speight, R., O’Hara, I., & Harrison, M. D. (2022). Filamentous fungi for future functional food and feed. Current Opinion in Biotechnology, 76, 102729. 10.1016/j.copbio.2022.102729 10.1016/j.copbio.2022.102729 35525176

[124] Finnigan, T. J. A., Wall, B. T., Wilde, P. J., Stephens, F. B., Taylor, S. L., & Freedman, M. R. (2019). Mycoprotein: The Future of Nutritious Nonmeat Protein, a Symposium Review. Current Developments in Nutrition, 3(6). 10.1093/cdn/nzz021 10.1093/cdn/nzz021 PMC6554455 31187084

[125] Jin, B., van Leeuwen, H. J., Patel, B., & Yu, Q. (1998). Utilisation of starch processing wastewater for production of microbial biomass protein and fungal α-amylase by Aspergillus oryzae. Bioresource Technology, 66(3), 201–206. 10.1016/S0960-8524(98)00060-1

[126] Serba, E., Pimenov, N., Mochalina, P., Overchenko, M., Borscheva, Y., Sharikov, A., & Rimareva, L. (2020). Production of Aspergillus oryzae RCAM 01133 biomass with increased protein and polysaccharides content using by-products of food industry. Agronomy Research, 18(1), 290–300. 10.15159/AR.20.026

[127] Razzaq ZU, Khan MKI, Maan AA, Rahman S, ur. Characterization of single cell protein from Saccharomyces cerevisiae for nutritional, functional and antioxidant properties. Journal of Food Measurement and Characterization. 2020;14(5):2520–8. 10.1007/s11694-020-00498-x.

[128] Cairns TC, Barthel L, Meyer V. Something old, something new: challenges and developments in Aspergillus niger biotechnology. Essays Biochem. 2021;65(2):213–24. 10.1042/EBC20200139. 33955461 10.1042/EBC20200139 PMC8314004

[129] Rousta N, Ferreira JA, Taherzadeh MJ. Production of L-carnitine-enriched edible filamentous fungal biomass through submerged cultivation. Bioengineered. 2021;12(1):358–68. 10.1080/21655979.2020.1863618. 33323030 10.1080/21655979.2020.1863618 PMC8806343

[130] Barker, T. W., Drouliscos, N. J., & Worgan, J. T. (1981). Composition and nutritional evaluation of Aspergillus oryzae biomass grown on palm oil processing effluents. Journal of the Science of Food and Agriculture, 32(10), 1014–1020. 10.1002/jsfa.2740321010

[131] Jach, M. E., Serefko, A., Ziaja, M., & Kieliszek, M. (2022). Yeast Protein as an Easily Accessible Food Source. Metabolites, 12(1). 10.3390/metabo12010063 10.3390/metabo12010063 PMC8780597 35050185

[132] Prosvirnikov, D., Tuntsev, D., Gizzatullina, L., Kulikova, Y., Michaud, P., & Babich, O. (2023). Protein production from cellulosic waste using candida utilis. Environmental Technology & Innovation, 32, 103445. 10.1016/j.eti.2023.103445

[133] Øvrum Hansen, J., Hofossæter, M., Sahlmann, C., Ånestad, R., Reveco-Urzua, F. E., Press, C. M., Mydland, L. T., & Øverland, M. (2019). Effect of Candida utilis on growth and intestinal health of Atlantic salmon (Salmo salar) parr. Aquaculture, 511, 734239. 10.1016/j.aquaculture.2019.734239

[134] Gao L, Chi Z, Sheng J, Ni X, Wang L. Single-cell protein production from Jerusalem artichoke extract by a recently isolated marine yeast Cryptococcus aureus G7a and its nutritive analysis. Appl Microbiol Biotechnol. 2007;77(4):825–32. 10.1007/s00253-007-1210-7. 17929010 10.1007/s00253-007-1210-7

[135] Zhang T, Chi Z, Sheng J. A Highly Thermosensitive and Permeable Mutant of the Marine Yeast Cryptococcus aureus G7a Potentially Useful for Single-Cell Protein Production and its Nutritive Components. Mar Biotechnol. 2009;11(2):280–6. 10.1007/s10126-008-9144-3. 10.1007/s10126-008-9144-3 18807088

[136] Coelho MOC, Monteyne AJ, Dunlop MV, Harris HC, Morrison DJ, Stephens FB, Wall BT. Mycoprotein as a possible alternative source of dietary protein to support muscle and metabolic health. Nutr Rev. 2020;78(6):486–97. 10.1093/nutrit/nuz077. 31841152 10.1093/nutrit/nuz077

[137] Bartholomai BM, Ruwe KM, Thurston J, Jha P, Scaife K, Simon R, Abdelmoteleb M, Goodman RE, Farhi M. Safety evaluation of Neurospora crassa mycoprotein for use as a novel meat alternative and enhancer. Food Chem Toxicol. 2022;168: 113342. 10.1016/J.FCT.2022.113342. 35963473 10.1016/j.fct.2022.113342

[138] Dundar, A., Acay, H., & Yildiz, A. (2009). Effect of using different lignocellulosic wastes for cultivation of Pleurotus ostreatus (Jacq.) P. Kumm. on mushroom yield, chemical composition and nutritional value. African Journal of Biotechnology, 8(4), 662–666. http://www.academicjournals.org/AJB

[139] Khan A, Tania M. Nutritional and Medicinal Importance of Pleurotus Mushrooms: An Overview. Food Rev Intl. 2012;28(3):313–29. 10.1080/87559129.2011.637267.

[140] Pelaes Vital, A. C., Goto, P. A., Hanai, L. N., Gomes-da-Costa, S. M., de Abreu Filho, B. A., Nakamura, C. V., & Matumoto-Pintro, P. T. (2015). Microbiological, functional and rheological properties of low fat yogurt supplemented with Pleurotus ostreatus aqueous extract. LWT - Food Science and Technology, 64(2), 1028–1035. 10.1016/j.lwt.2015.07.003

[141] Yamada EA, Sgarbieri VC. Yeast (Saccharomyces cerevisiae) Protein Concentrate: Preparation, Chemical Composition, and Nutritional and Functional Properties. J Agric Food Chem. 2005;53(10):3931–6. 10.1021/jf0400821. 15884819 10.1021/jf0400821

[142] Soccol, C. R., Costa, E. S. F. da, Letti, L. A. J., Karp, S. G., Woiciechowski, A. L., & Vandenberghe, L. P. de S. (2017). Recent developments and innovations in solid state fermentation. Biotechnology Research and Innovation, 1(1), 52–71. 10.1016/j.biori.2017.01.002

[143] Samadi, S., Mohammadi, M., & Najafpour, G. D. (2016). Production of Single Cell Protein from Sugarcane Bagasse by Saccharomyces cerevisiae in Tray Bioreactor. International Journal of Engineering, 29(8). 10.5829/idosi.ije.2016.29.08b.01

[144] Khalil MI, Hossain S, Khalil MI, Alam MK, Khan MA, Alam N. Upgrading of Animal Feed by Solid State Fermentation by Pleurotus sajor-caju. European Journal of Applied Sciences. 2009;1(4):53–8.

[145] Lourens, V. (2023). Pre-treatment of sugarcane bagasse for conversion to single cell protein through one-step enzymatic hydrolysis and bioconversion. https://scholar.sun.ac.za

[146] Zhao, S., Wang, Z. B., Wang, Y. C., Yang, P. Y., Luo, X. M., Wu, A. M., & Feng, J. X. (2023). Sustainable coproduction of xylooligosaccharide, single-cell protein and lignin-adsorbent through whole components’ utilization of sugarcane bagasse with high solid loading. Separation and Purification Technology, 308. 10.1016/j.seppur.2022.122916

[147] Sun, W., Zhang, Z., Li, X., Lu, X., Liu, G., Qin, Y., Zhao, J., & Qu, Y. (2024a). Production of single cell protein from brewer’s spent grain through enzymatic saccharification and fermentation enhanced by ammoniation pretreatment. Bioresource Technology, 394. 10.1016/j.biortech.2023.130242 10.1016/j.biortech.2023.130242 38145760

[148] Nair RB, Taherzadeh MJ. Valorization of sugar-to-ethanol process waste vinasse: A novel biorefinery approach using edible ascomycetes filamentous fungi. Biores Technol. 2016;221:469–76. 10.1016/j.biortech.2016.09.074. 10.1016/j.biortech.2016.09.074 27668880

[149] Nitayavardhana S, Issarapayup K, Pavasant P, Khanal SK. Production of protein-rich fungal biomass in an airlift bioreactor using vinasse as substrate. Biores Technol. 2013;133:301–6. 10.1016/j.biortech.2013.01.073. 10.1016/j.biortech.2013.01.073 23434806

[150] Nigam JN. Cultivation of Candida langeronii in sugar cane bagasse hemicellulosic hydrolyzate for the production of single cell protein. J Biotechnol. 2000;51(1):83–8. 10.1016/0168-1656(96)01572-6.

[151] Nigam JN. Single cell protein from pineapple cannery effluent. World J Microbiol Biotechnol. 1998;14(5):693–6. 10.1023/A:1008853303596.

[152] Wiebe M. Myco-protein from Fusarium venenatum: a well-established product for human consumption. Appl Microbiol Biotechnol. 2002;58(4):421–7. 10.1007/s00253-002-0931-x. 11954786 10.1007/s00253-002-0931-x

[153] Seyed Reihani, S. F., & Khosravi-Darani, K. (2019). Mycoprotein Production from Date Waste Using Fusarium venenatum in a Submerged Culture. Applied Food Biotechnology, 5(4), 243–352. 10.22037/afb.v5i4.23139

[154] Saejung C, Thammaratana T. Biomass recovery during municipal wastewater treatment using photosynthetic bacteria and prospect of production of single cell protein for feedstuff. Environ Technol. 2016;37(23):3055–61. 10.1080/09593330.2016.1175512. 27070497 10.1080/09593330.2016.1175512

[155] Shipman, R. H., Kao, I. C., & Fan, L. T. (1975). Single-cell protein production by photosynthetic bacteria cultivation in agricultural by-products. Biotechnology and Bioengineering, 17(11), 1561–1570. 10.1002/bit.260171102

[156] Bacha U, Nasir M, Khalique A, Anjum AA, Jabbar MA. Comparative assessment of various agro-industrial wastes for saccharomyces cerevisiae biomass production and its quality evaluation as single cell protein. J Anim Plant Sci. 2011;21(4):844–9.

[157] Shi C, He J, Yu J, Yu B, Huang Z, Mao X, Zheng P, Chen D. Solid state fermentation of rapeseed cake with Aspergillus niger for degrading glucosinolates and upgrading nutritional value. Journal of Animal Science and Biotechnology. 2015;6(1):13. 10.1186/s40104-015-0015-2. 25883784 10.1186/s40104-015-0015-2 PMC4399751

[158] Chawla, P., Bhandari, L., Sadh, P. K., & Kaushik, R. (2017). Impact of Solid-State Fermentation (Aspergillus oryzae) on Functional Properties and Mineral Bioavailability of Black-Eyed Pea (Vigna unguiculata) Seed Flour. Cereal Chemistry, 94(3), 437–442. 10.1094/CCHEM-05-16-0128-R

[159] Yunus F, un N., Nadeem, M., & Rashid, F. Single-cell protein production through microbial conversion of lignocellulosic residue (wheat bran) for animal feed. J Inst Brew. 2015;121(4):553–7. 10.1002/jib.251.

[160] Gmoser, R., Sintca, C., Taherzadeh, M. J., & Lennartsson, P. R. (2019). Combining submerged and solid state fermentation to convert waste bread into protein and pigment using the edible filamentous fungus N. intermedia. Waste Management, 97, 63–70. 10.1016/j.wasman.2019.07.039 10.1016/j.wasman.2019.07.039 31447028

[161] Sitanggang AB, Sinaga WSL, Wie F, Fernando F, Krusong W. Enhanced antioxidant activity of okara through solid state fermentation of GRAS fungi. Food Science and Technology (Brazil). 2020;40(1):178–86. 10.1590/fst.37218.

[162] Heidari, F., Øverland, M., Hansen, J. Ø., Mydland, L. T., Urriola, P. E., Chen, C., Shurson, G. C., & Hu, B. (2022). Solid-state fermentation of Pleurotus ostreatus to improve the nutritional profile of mechanically-fractionated canola meal. Biochemical Engineering Journal, 187, 108591. 10.1016/j.bej.2022.108591

[163] Almeida, J. R. M., Modig, T., Petersson, A., Hähn-Hägerdal, B., Lidén, G., & Gorwa-Grauslund, M. F. (2007). Increased tolerance and conversion of inhibitors in lignocellulosic hydrolysates by Saccharomyces cerevisiae. In Journal of Chemical Technology and Biotechnology (Vol. 82, Issue 4). 10.1002/jctb.1676

[164] Palmqvist, E., & Hahn-Hägerdal, B. (2000). Fermentation of lignocellulosic hydrolysates. I: Inhibition and detoxification. Bioresource Technology, 74(1). 10.1016/S0960-8524(99)00160-1

[165] Wheals, A. E., Basso, L. C., Alves, D. M. G., & Amorim, H. V. (1999). Fuel ethanol after 25 years. In Trends in Biotechnology (Vol. 17, Issue 12). 10.1016/S0167-7799(99)01384-0 10.1016/s0167-7799(99)01384-0 10557161

[166] Max B, Salgado JM, Rodríguez N, Cortés S, Converti A, Manuel Domínguez J. BIOTECHNOLOGICAL PRODUCTION OF CITRIC ACID. Braz J Microbiol. 2010;41:862–75. 24031566 10.1590/S1517-83822010000400005 PMC3769771

[167] Guimarães, P. M. R., Teixeira, J. A., & Domingues, L. (2010). Fermentation of lactose to bio-ethanol by yeasts as part of integrated solutions for the valorisation of cheese whey. In Biotechnology Advances (Vol. 28, Issue 3). 10.1016/j.biotechadv.2010.02.002 10.1016/j.biotechadv.2010.02.002 20153415

[168] Wang, Z., & Yang, S. T. (2013). Propionic acid production in glycerol/glucose co-fermentation by Propionibacterium freudenreichii subsp. shermanii. Bioresource Technology, 137. 10.1016/j.biortech.2013.03.012 10.1016/j.biortech.2013.03.012 23584412

[169] Jones, D. T., & Woods, D. R. (1986). Acetone-Butanol Fermentation Revisited. In MICROBIOLOGICAL REVIEWS (Vol. 50, Issue 4). 10.1128/mr.50.4.484-524.1986 PMC373084 3540574

[170] Hofvendahl, K., & Hahn-Hägerdal, B. (2000). Factors affecting the fermentative lactic acid production from renewable resources. Enzyme and Microbial Technology, 26(2–4). 10.1016/S0141-0229(99)00155-6 10.1016/s0141-0229(99)00155-6 10689064

[171] Gupta, R., Beg, Q. K., Khan, S., & Chauhan, B. (2002). An overview on fermentation, downstream processing and properties of microbial alkaline proteases. In Applied Microbiology and Biotechnology (Vol. 60, Issue 4). 10.1007/s00253-002-1142-1 10.1007/s00253-002-1142-1 12466877

[172] Ravindra, P. (2000). Value-added food: Single cell protein. In Biotechnology Advances (Vol. 18). 10.1016/s0734-9750(00)00045-8 14538097

[173] Kim S, Dale BE. Global potential bioethanol production from wasted crops and crop residues. Biomass Bioenerg. 2004;26(4):361–75. 10.1016/j.biombioe.2003.08.002.

[174] Kampen, W. H. (2014). Nutritional Requirements in Fermentation Processes. Fermentation and Biochemical Engineering Handbook: Principles, Process Design, and Equipment: Third Edition, 37–57. 10.1016/B978-1-4557-2553-3.00004-0

[175] Roca-Mesa H, Delgado-Yuste E, Mas A, Torija MJ, Beltran G. Importance of micronutrients and organic nitrogen in fermentations with Torulaspora delbrueckii and Saccharomyces cerevisiae. Int J Food Microbiol. 2022;381: 109915. 10.1016/J.IJFOODMICRO.2022.109915. 36084391 10.1016/j.ijfoodmicro.2022.109915

[176] Walker, G. M. (1994). The roles of magnesium in biotechnology. Critical Reviews in Biotechnology, 14(4). 10.3109/07388559409063643 10.3109/07388559409063643 7889576

[177] Mussatto, S. I. (2014). Brewer’s spent grain: A valuable feedstock for industrial applications. Journal of the Science of Food and Agriculture, 94(7). 10.1002/jsfa.6486 10.1002/jsfa.6486 24254316

[178] Zhao XQ, Bai F, wu. Zinc and yeast stress tolerance: Micronutrient plays a big role. J Biotechnol. 2012;158(4):176–83. 10.1016/j.jbiotec.2011.06.038. 21763361 10.1016/j.jbiotec.2011.06.038

[179] Yadav, M., Goswami, P., Paritosh, K., Kumar, M., Pareek, N., & Vivekanand, V. (2019). Seafood waste: a source for preparation of commercially employable chitin/chitosan materials. In Bioresources and Bioprocessing (Vol. 6, Issue 1). 10.1186/s40643-019-0243-y

[180] Lin, Y., & Tanaka, S. (2006). Ethanol fermentation from biomass resources: Current state and prospects. In Applied Microbiology and Biotechnology (Vol. 69, Issue 6). 10.1007/s00253-005-0229-x 10.1007/s00253-005-0229-x 16331454

[181] Ren, X., Wang, J., Yu, H., Peng, C., Hu, J., Ruan, Z., Zhao, S., Liang, Y., & Peng, N. (2016). Anaerobic and sequential aerobic production of high-titer ethanol and single cell protein from NaOH-pretreated corn stover by a genome shuffling-modified Saccharomyces cerevisiae strain. Bioresource Technology, 218, 623–630. 10.1016/j.biortech.2016.06.118 10.1016/j.biortech.2016.06.118 27416512

[182] Khanifar J, Ghoorchian H, Ahmadi AR, Hajihosaini R. Comparison of essential and non essential amino acids in the single cell protein (scp) of white rot fungi from wheat straw. Afr J Agric Res. 2011;6(17):3994–9. 10.5897/AJAR11.830.

[183] Zaki, M., & Said, S. D. (2018). Trichoderma Reesei single cell protein production from rice straw pulp in solid state fermentation. IOP Conference Series: Materials Science and Engineering, 345(1). 10.1088/1757-899X/345/1/012043

[184] Amaro Bittencourt, G., Porto de Souza Vandenberghe, L., Valladares-Diestra, K., Wedderhoff Herrmann, L., Fátima Murawski de Mello, A., Sarmiento Vásquez, Z., Grace Karp, S., & Ricardo Soccol, C. (2021). Soybean hulls as carbohydrate feedstock for medium to high-value biomolecule production in biorefineries: A review. Bioresource Technology, 339, 125594. 10.1016/j.biortech.2021.125594 10.1016/j.biortech.2021.125594 34311407

[185] Zhong, P., Chen, P., Huo, P., Ma, L., Xu, Z., Li, F., & Cai, C. (2025). Characterization of cotton stalk as a lignocellulosic feedstock for single-cell protein production. Bioresource Technology, 417, 131797. 10.1016/j.biortech.2024.131797 10.1016/j.biortech.2024.131797 39580094

[186] Najari, Z., Khodaiyan, F., Yarmand, M. S., & Hosseini, S. S. (2022). Almond hulls waste valorization towards sustainable agricultural development: Production of pectin, phenolics, pullulan, and single cell protein. Waste Management, 141, 208–219. 10.1016/j.wasman.2022.01.007 10.1016/j.wasman.2022.01.007 35149477

[187] Pruksasri, S., Wollinger, K. K., & Novalin, S. (2019). Transformation of rice bran into single-cell protein, extracted protein, soluble and insoluble dietary fiber, and minerals. Journal of the Science of Food and Agriculture, 99(11), 5044–5049. 10.1002/jsfa.9747 10.1002/jsfa.9747 30980414

[188] Sun, W., Zhang, Z., Li, X., Lu, X., Liu, G., Qin, Y., Zhao, J., & Qu, Y. (2024b). Production of single cell protein from brewer’s spent grain through enzymatic saccharification and fermentation enhanced by ammoniation pretreatment. Bioresource Technology, 394, 130242. 10.1016/j.biortech.2023.130242 10.1016/j.biortech.2023.130242 38145760

[189] Parchami, M., Mahboubi, A., Agnihotri, S., & Taherzadeh, M. J. (2023). Biovalorization of brewer’s spent grain as single-cell protein through coupling organosolv pretreatment and fungal cultivation. Waste Management, 169, 382–391. 10.1016/j.wasman.2023.07.021 10.1016/j.wasman.2023.07.021 37531932

[190] Hashem M, Al-Qahtani MS, Alamri SA, Moustafa YS, Lyberatos G, Ntaikou I. Valorizing food wastes: assessment of novel yeast strains for enhanced production of single-cell protein from wasted date molasses. Biomass Conversion and Biorefinery. 2022;12(10):4491–502. 10.1007/s13399-022-02415-2.

[191] Nigam, P., & Vogel, M. (1991). Bioconversion of sugar industry by-products—molasses and sugar beet pulp for single cell protein production by yeasts. Biomass and Bioenergy, 1(6), 339–345. 10.1016/0961-9534(91)90014-4

[192] Thiviya, P., Gamage, A., Kapilan, R., Merah, O., & Madhujith, T. (2022). Single Cell Protein Production Using Different Fruit Waste: A Review. In Separations (Vol. 9, Issue 7). MDPI. 10.3390/separations9070178

[193] Sharma, S., Sindhu, S., Saloni, S., & Singh, P. (2024). Chapter 11 - Production of singlecell protein from fruit wastes. In S. P. Bangar & P. S. Panesar (Eds.), Adding Value to Fruit Wastes (pp. 291–313). Academic Press. 10.1016/B978-0-443-13842-3.00011-3

[194] Khan, M. U., & Ahring, B. K. (2019). Lignin degradation under anaerobic digestion: Influence of lignin modifications - A review. Biomass and Bioenergy, 128, 105325. 10.1016/j.biombioe.2019.105325

[195] Segers, B., Nimmegeers, P., Spiller, M., Tofani, G., Jasiukaitytė-Grojzdek, E., Dace, E., Kikas, T., Marchetti, J. M., Rajić, M., Yildiz, G., & Billen, P. (2024). Lignocellulosic biomass valorisation: a review of feedstocks, processes and potential value chains and their implications for the decision-making process. RSC Sustainability, 2(12), 3730–3749. 10.1039/d4su00342j

[196] Hendriks, A. T. W. M., & Zeeman, G. (2009). Pretreatments to enhance the digestibility of lignocellulosic biomass. In Bioresource Technology (Vol. 100, Issue 1, pp. 10–18). Elsevier Ltd. 10.1016/j.biortech.2008.05.027 10.1016/j.biortech.2008.05.027 18599291

[197] Arce, C., & Kratky, L. (2022). Mechanical pretreatment of lignocellulosic biomass toward enzymatic/fermentative valorization. IScience, 25(7), 104610. 10.1016/j.isci.2022.104610 10.1016/j.isci.2022.104610 PMC9250023 35789853

[198] Duque, A., Manzanares, P., González, A., & Ballesteros, M. (2018). Study of the Application of Alkaline Extrusion to the Pretreatment of Eucalyptus Biomass as First Step in a Bioethanol Production Process. Energies, 11(11). 10.3390/en11112961

[199] Bussemaker MJ, Zhang D. Effect of Ultrasound on Lignocellulosic Biomass as a Pretreatment for Biorefinery and Biofuel Applications. Ind Eng Chem Res. 2013;52(10):3563–80. 10.1021/ie3022785.

[200] Zhu H, Cheng JH, Ma J, Sun DW. Deconstruction of pineapple peel cellulose based on Fe2+ assisted cold plasma pretreatment for cellulose nanofibrils preparation. Food Chem. 2023;401: 134116. 10.1016/J.FOODCHEM.2022.134116. 36113216 10.1016/j.foodchem.2022.134116

[201] Lin SP, Kuo TC, Wang HT, Ting Y, Hsieh CW, Chen YK, Hsu HY, Cheng KC. Enhanced bioethanol production using atmospheric cold plasma-assisted detoxification of sugarcane bagasse hydrolysate. Biores Technol. 2020;313: 123704. 10.1016/J.BIORTECH.2020.123704. 10.1016/j.biortech.2020.123704 32590306

[202] Park YC, Kim JS. Comparison of various alkaline pretreatment methods of lignocellulosic biomass. Energy. 2012. 10.1016/j.energy.2012.08.010.

[203] Kim, J. S., Lee, Y. Y., & Kim, T. H. (2016). A review on alkaline pretreatment technology for bioconversion of lignocellulosic biomass. In Bioresource Technology (Vol. 199, pp. 42–48). Elsevier Ltd. 10.1016/j.biortech.2015.08.085 10.1016/j.biortech.2015.08.085 26341010

[204] de Moraes J, Rocha G, Martin C, Soares IB, Souto Maior AM, Baudel HM, Moraes de Abreu CA. Dilute mixed-acid pretreatment of sugarcane bagasse for ethanol production. Biomass Bioenerg. 2011;35(1):663–70. 10.1016/j.biombioe.2010.10.018.

[205] Sritrakul, N., Nitisinprasert, S., & Keawsompong, S. (2017). Evaluation of dilute acid pretreatment for bioethanol fermentation from sugarcane bagasse pith. Agriculture and Natural Resources, 51(6), 512–519. 10.1016/j.anres.2017.12.006

[206] Sidiras, D., Politi, D., Giakoumakis, G., & Salapa, I. (2022). Simulation and optimization of organosolv based lignocellulosic biomass refinery: A review. Bioresource Technology, 343, 126158. 10.1016/j.biortech.2021.126158 10.1016/j.biortech.2021.126158 34673192

[207] Zhou, Z., Ouyang, D., Liu, D., & Zhao, X. (2023). Oxidative pretreatment of lignocellulosic biomass for enzymatic hydrolysis: Progress and challenges. Bioresource Technology, 367, 128208. 10.1016/j.biortech.2022.128208 10.1016/j.biortech.2022.128208 36323374

[208] Jin, M., & Dale, B. E. (2018). AFEX^TM^ Pretreatment-Based Biorefinery Technologies. In J. M. Park (Ed.), Handbook of Biorefinery Research and Technology (pp. 1–16). Springer Netherlands. 10.1007/978-94-007-6724-9_2-2

[209] Balan, V., Bals, B., Chundawat, S. P. S., Marshall, D., & Dale, B. E. (2009). Lignocellulosic Biomass Pretreatment Using AFEX. In J. R. Mielenz (Ed.), Biofuels: Methods and Protocols (pp. 61–77). Humana Press. 10.1007/978-1-60761-214-8_5 10.1007/978-1-60761-214-8_5 19768616

[210] Mokomele T, da Costa Sousa L, Bals B, Balan V, Goosen N, Dale BE, Görgens JF. Using steam explosion or AFEX™ to produce animal feeds and biofuel feedstocks in a biorefinery based on sugarcane residues. Biofuels, Bioprod Biorefin. 2018;12(6):978–96. 10.1002/bbb.1927.

[211] Yu, Y., Wu, J., Ren, X., Lau, A., Rezaei, H., Takada, M., Bi, X., & Sokhansanj, S. (2022). Steam explosion of lignocellulosic biomass for multiple advanced bioenergy processes: A review. Renewable and Sustainable Energy Reviews, 154, 111871. 10.1016/j.rser.2021.111871

[212] Wang W, Zhuang X, Yuan Z, Yu Q, Qi W, Wang Q, Tan X. High consistency enzymatic saccharification of sweet sorghum bagasse pretreated with liquid hot water. Biores Technol. 2012;108:252–7. 10.1016/j.biortech.2011.12.092. 10.1016/j.biortech.2011.12.092 22281144

[213] Toor, S. S., Rosendahl, L., & Rudolf, A. (2011). Hydrothermal liquefaction of biomass: A review of subcritical water technologies. In Energy (Vol. 36, Issue 5, pp. 2328–2342). 10.1016/j.energy.2011.03.013

[214] Sharma HK, Xu C, Qin W. Biological Pretreatment of Lignocellulosic Biomass for Biofuels and Bioproducts: An Overview. Waste and Biomass Valorization. 2019;10(2):235–51. 10.1007/s12649-017-0059-y.

[215] Rebitzer G, Ekvall T, Frischknecht R, Hunkeler D, Norris G, Rydberg T, Schmidt WP, Suh S, Weidema BP, Pennington DW. Life cycle assessment: Part 1: Framework, goal and scope definition, inventory analysis, and applications. Environ Int. 2004;30(5):701–20. 10.1016/J.ENVINT.2003.11.005. 15051246 10.1016/j.envint.2003.11.005

[216] Sagar NA, Pathak M, Sati H, Agarwal S, Pareek S. Advances in pretreatment methods for the upcycling of food waste: A sustainable approach. Trends Food Sci Technol. 2024;147: 104413. 10.1016/J.TIFS.2024.104413.

[217] da Fonseca YA, Fernandes ARAC, Gurgel LVA, Baêta BEL. Comparative life cycle assessment of early-stage technological layouts for brewers’ spent grain upcycling: A sustainable approach for adding value to waste. Journal of Water Process Engineering. 2024;66: 105904. 10.1016/J.JWPE.2024.105904.

[218] Ferreira, H., Silva, S., Silva, C., Oliveira, S., Ribeiro, A. B., Nutrizio, M., Duki´cduki´c, J., Sabljak, I., Samardžija, A., Biondi´c, V., Fučkar, B., Djeki´c, I. D., & Jambrak, A. R. (2024). Upcycling of Food By-Products and Waste: Nonthermal Green Extractions and Life Cycle Assessment Approach. Sustainability 2024, Vol. 16, Page 9143, 16(21), 9143. 10.3390/SU16219143

[219] Järviö, N., Parviainen, T., Maljanen, N. L., Kobayashi, Y., Kujanpää, L., Ercili-Cura, D., Landowski, C. P., Ryynänen, T., Nordlund, E., & Tuomisto, H. L. (2021). Ovalbumin production using Trichoderma reesei culture and low-carbon energy could mitigate the environmental impacts of chicken-egg-derived ovalbumin. Nature Food, 2(12). 10.1038/s43016-021-00418-2 10.1038/s43016-021-00418-2 37118250

[220] Sinke, P., Swartz, E., Sanctorum, H., van der Giesen, C., & Odegard, I. (2023). Ex-ante life cycle assessment of commercial-scale cultivated meat production in 2030. International Journal of Life Cycle Assessment, 28(3). 10.1007/s11367-022-02128-8

[221] Chezan D, Flannery O, Patel A. Factors affecting consumer attitudes to fungi-based protein: A pilot study. Appetite. 2022;175: 106043. 10.1016/J.APPET.2022.106043. 35487309 10.1016/j.appet.2022.106043

[222] Quorn. (2025, January 14). Quorn. https://Www.Quorn.Us/.

[223] Enough. (2025, January 10). Enough. Delicious. Nutritious. Sustainable. https://Www.Enough-Food.Com/.

[224] Jones, N. (2023). Fungi bacon and insect burgers: a guide to the proteins of the future. In Nature (Vol. 619, Issue 7968). 10.1038/d41586-023-02096-5

[225] Dean, D., Rombach, M., Koning, W. de, Vriesekoop, F., Satyajaya, W., Yuliandari, P., Anderson, M., Mongondry, P., Urbano, B., Luciano, C. A. G., Hao, W., Eastwick, E., Achirimbi, E., Jiang, Z., Boereboom, A., Rashid, F., Khan, I., Alvarez, B., & Aguiar, L. K. (2022). Understanding Key Factors Influencing Consumers’ Willingness to Try, Buy, and Pay a Price Premium for Mycoproteins. Nutrients, 14(16). 10.3390/nu14163292 10.3390/nu14163292 PMC9416216 36014797

[226] Whittaker, J. A., Johnson, R. I., Finnigan, T. J. A., Avery, S. V., & Dyer, P. S. (2020). The Biotechnology of Quorn Mycoprotein: Past, Present and Future Challenges. In Grand Challenges in Biology and Biotechnology. 10.1007/978-3-030-29541-7_3

[227] Wiebe, M. G. (2004). Quorn^TM^ myco-protein - Overview of a successful fungal product. In Mycologist (Vol. 18, Issue 1, pp. 17–20). Cambridge University Press. 10.1017/S0269915X04001089

[228] The Better Meat Co. (2025, January 17). The Better Meat Co. - Mycoprotein Ingredients for Better Meats. https://Www.Bettermeat.Co/.

[229] Meati. (2025, January 17). Eat Meati. https://Www.Meati.Com/.

[230] Caminiti J, Badiger A, Amoafo O, Serventi L. Understanding New Foods: Alternative Protein Sources. Sustainable Development Goals Series, Part. 2023;F2751:135–46. 10.1007/978-3-031-12358-0_10.

[231] Finnigan TJA, Theobald HE, Bajka B. Mycoprotein: A Healthy and Sustainable Source of Alternative Protein-Based Foods. Annu Rev Food Sci Technol. 2024. 10.1146/ANNUREV-FOOD-111523-121802. 10.1146/annurev-food-111523-121802 39626232

[232] Furlan, O., de Oliveira, N. S., de Paula, R. C., Rosa, R. T., Michelotto, P. V., Weber, S. H., Bianchini, L. F., & Rosa, E. A. R. (2024). Pilot scale production of high-content mycoprotein using Rhizopus microsporus var. oligosporus by submerged fermentation and agro-industrial by-products. Bioresource Technology, 413, 131515. 10.1016/J.BIORTECH.2024.131515 10.1016/j.biortech.2024.131515 39366513

[233] Michelin M, de Oliveira Mota AM, de Polizeli M, L. T. de M., da Silva, D. P., Vicente, A. A., & Teixeira, J. A. Influence of volumetric oxygen transfer coefficient (kLa) on xylanases batch production by Aspergillus niger van Tieghem in stirred tank and internal-loop airlift bioreactors. Biochem Eng J. 2013;80:19–26. 10.1016/J.BEJ.2013.09.002.

[234] Perkins DD, Davis RH. Neurospora at the millennium. Fungal Genet Biol. 2000;31(3):153–67. 10.1006/fgbi.2000.1248. 11273678 10.1006/fgbi.2000.1248

[235] Beadle, G. W., & Tatum, E. L. (1941). Genetic Control of Biochemical Reactions in Neurospora. Proceedings of the National Academy of Sciences, 27(11). 10.1073/pnas.27.11.499 10.1073/pnas.27.11.499 PMC1078370 16588492

[236] Enifer. (2025, January 17). Ingredients for a sustainable food chain. https://Enifer.Com/.

[237] Hooft JM, Tran HQ, Montero R, Morales-Lange B, Stejskal V, Mydland LT, Øverland M. Environmental impacts of the filamentous fungi Paecilomyces variotii (PEKILO®) as a novel protein source in feeds for Atlantic salmon (Salmo salar). Aquaculture. 2025;596: 741779. 10.1016/J.AQUACULTURE.2024.741779.

[238] MyForest Foods. (2025, January 20). FARMED Not FAKE. https://Myforestfoods.Com/.

[239] Mycorena. (2025, January 17). Promyc. https://Www.Promyc.Com/.

[240] Mycovation. (2025, January 18). Mycovation. https://Www.Mycovation.Asia/.

[241] Aqua Cultured Foods. (2025, January 20). Aqua Cultured Foods. https://Www.Aquaculturedfoods.Com/.

[242] Kidemis. (2025, January 21). Sustainable Biotechnology to Feed the Planet. https://Www.Kidemis.Com/.

[243] Funki. (2025, January 17). Rooted in mycoprotein. https://Www.Funki.Ee/.

[244] Libre Foods. (2025, January 19). REINVENTING FOOD WITH FUNGI. https://Www.Librefoods.Co/.

[245] Tempty Foods. (2025, January 16). TEMPTY is a prize-winning alternative. https://Www.Tempty-Foods.Com/.

[246] Souza Filho, P. F., Andersson, D., Ferreira, J. A., & Taherzadeh, M. J. (2019). Mycoprotein: environmental impact and health aspects. In World Journal of Microbiology and Biotechnology (Vol. 35, Issue 10). 10.1007/s11274-019-2723-9 10.1007/s11274-019-2723-9 PMC6757021 31549247

[247] Seyed Reihani, S. F., & Khosravi-Darani, K. (2018). Mycoprotein production from date waste using Fusarium venenatum in a submerged culture. Applied Food Biotechnology, 5(4). 10.22037/afb.v%vi%i.23139

[248] Finnigan, T., Needham, L., & Abbott, C. (2016). Mycoprotein: A Healthy New Protein With a Low Environmental Impact. In Sustainable Protein Sources (pp. 305–325). Elsevier Inc. 10.1016/B978-0-12-802778-3.00019-6

[249] Pradal, D., Vauchel, P., Decossin, S., Dhulster, P., & Dimitrov, K. (2016). Kinetics of ultrasound-assisted extraction of antioxidant polyphenols from food by-products: Extraction and energy consumption optimization. Ultrasonics Sonochemistry, 32. 10.1016/j.ultsonch.2016.03.001 10.1016/j.ultsonch.2016.03.001 27150754

[250] Suchintita Das, R., Tiwari, B. K., Chemat, F., & Garcia-Vaquero, M. (2022). Impact of ultrasound processing on alternative protein systems: Protein extraction, nutritional effects and associated challenges. In Ultrasonics Sonochemistry (Vol. 91). 10.1016/j.ultsonch.2022.106234 10.1016/j.ultsonch.2022.106234 PMC9685360 36435088

[251] Jiang, G., Ameer, K., Kim, H., Lee, E. J., Ramachandraiah, K., & Hong, G. P. (2020). Strategies for sustainable substitution of livestock meat. In Foods (Vol. 9, Issue 9). MDPI AG. 10.3390/foods9091227 10.3390/foods9091227 PMC7555167 32899106

[252] Hashempour-Baltork F, Jannat B, Dadgarnejad M, Mirza Alizadeh A, Khosravi-Darani K, Hosseini H. Mycoprotein as chicken meat substitute in nugget formulation: Physicochemical and sensorial characterization. Food Sci Nutr. 2023. 10.1002/fsn3.3354. 10.1002/fsn3.3354 PMC10345705 37457149

[253] Crater, J. S., & Lievense, J. C. (2018). Scale-up of industrial microbial processes. FEMS Microbiology Letters, 365(13), fny138. 10.1093/femsle/fny138 10.1093/femsle/fny138 PMC5995164 29860483

[254] Surya Ulhas, R., Ravindran, R., Malaviya, A., Priyadarshini, A., Tiwari, B. K., & Rajauria, G. (2023). A review of alternative proteins for vegan diets: Sources, physico-chemical properties, nutritional equivalency, and consumer acceptance. Food Research International, 173. 10.1016/j.foodres.2023.113479 10.1016/j.foodres.2023.113479 37803803

[255] Abou-Zeid, A.-Z. A., Khan, J. A., & Abulnaja, K. O. (1995). On methods for reduction of nucleic acids content in a single-cell protein from gas oil. *Bioresource Technology*, *52*(1), 21–24. 10.1016/0960-8524(95)99782-Q

[256] Calton, A., Lille, M., & Sozer, N. (2023). 3-D printed meat alternatives based on pea and single cell proteins and hydrocolloids: Effect of paste formulation on process-induced fibre alignment and structural and textural properties. Food Research International, 174, 113633. 10.1016/j.foodres.2023.113633 10.1016/j.foodres.2023.113633 37981359

[257] Chen BY, Chen SW, Wang HT. Use of different alkaline pretreatments and enzyme models to improve low-cost cellulosic biomass conversion. Biomass Bioenerg. 2012. 10.1016/j.biombioe.2012.01.012.

[258] Paz-Cedeno, F. R., Henares, L. R., Solorzano-Chavez, E. G., Scontri, M., Picheli, F. P., Miranda Roldán, I. U., Monti, R., Conceição de Oliveira, S., & Masarin, F. (2021). Evaluation of the effects of different chemical pretreatments in sugarcane bagasse on the response of enzymatic hydrolysis in batch systems subject to high mass loads. Renewable Energy, 165. 10.1016/j.renene.2020.10.092

